# Allogeneic CAR T Cells: An Alternative to Overcome Challenges of CAR T Cell Therapy in Glioblastoma

**DOI:** 10.3389/fimmu.2021.640082

**Published:** 2021-03-03

**Authors:** Darel Martínez Bedoya, Valérie Dutoit, Denis Migliorini

**Affiliations:** ^1^Center for Translational Research in Onco-Hematology, University of Geneva, Geneva, Switzerland; ^2^Swiss Cancer Center Léman, Lausanne, Switzerland; ^3^Brain Tumor and Immune Cell Engineering Group, Faculty of Medicine, University of Geneva, Geneva, Switzerland; ^4^Department of Oncology, Geneva University Hospitals (HUG), Geneva, Switzerland

**Keywords:** CAR T cells, glioblastoma, allogeneic, graft-vs.-host disease, allorejection, tumor microenvironment

## Abstract

Chimeric antigen receptor (CAR) T cell therapy has emerged as one of the major breakthroughs in cancer immunotherapy in the last decade. Outstanding results in hematological malignancies and encouraging pre-clinical anti-tumor activity against a wide range of solid tumors have made CAR T cells one of the most promising fields for cancer therapies. CAR T cell therapy is currently being investigated in solid tumors including glioblastoma (GBM), a tumor for which survival has only modestly improved over the past decades. CAR T cells targeting EGFRvIII, Her2, or IL-13Rα2 have been tested in GBM, but the first clinical trials have shown modest results, potentially due to GBM heterogeneity and to the presence of an immunosuppressive microenvironment. Until now, the use of autologous T cells to manufacture CAR products has been the norm, but this approach has several disadvantages regarding production time, cost, manufacturing delay and dependence on functional fitness of patient T cells, often reduced by the disease or previous therapies. Universal “off-the-shelf,” or allogeneic, CAR T cells is an alternative that can potentially overcome these issues, and allow for multiple modifications and CAR combinations to target multiple tumor antigens and avoid tumor escape. Advances in genome editing tools, especially *via* CRISPR/Cas9, might allow overcoming the two main limitations of allogeneic CAR T cells product, i.e., graft-vs.-host disease and host allorejection. Here, we will discuss how allogeneic CAR T cells could allow for multivalent approaches and alteration of the tumor microenvironment, potentially allowing the development of next generation therapies for the treatment of patients with GBM.

## Introduction

Chimeric antigen receptors (CAR) are synthetic receptors comprising an extracellular domain, most frequently derived from an antibody single-chain variable fragment (scFv), and an intracellular signaling and costimulatory domain derived from T cells. Genetic insertion of CARs, most frequently into the T cell genome but also in other immune cells, allows redirecting them to a desired antigen (1). Anti-CD19 CAR T cells, mainly generated from autologous peripheral blood lymphocytes, have shown remarkable clinical responses in patients with B cell-derived hematologic malignancies (2). Clinical trials using anti-CD19 CAR T cells led to a paradigm change in cancer therapy, based on their unprecedented response rates in adult patients with recurrent/refractory diffuse large B cell lymphoma (DLBCL) or pediatric refractory B cell acute lymphoblastic leukemia (B-ALL) (3–6). Two CAR T cell products specific for the B-cell marker CD19, Kymriah (Novartis) and Yescarta (Kite Pharma), became the first therapeutic products registered by the FDA comprising a genetic engineering element for the treatment of B-ALL and DLBCL (7, 8).

However, occurrence of severe side effects is associated with the use of CAR T cell therapy. The best-characterized toxicity associated with CAR T cells is cytokine-release syndrome (CRS). It consists of a systemic inflammatory response derived from immune cell activation, with common symptoms being the presence of hypotension, capillary leak, high fever and multiorgan failure (9, 10). CRS is produced by a supra-physiological activation of CAR T cells that leads to exacerbated secretion of pro-inflammatory cytokines, including IFN-γ, TNF-α, IL-6, and IL-2, and chemokines such as MCP1, allowing the recruitment and activation of other immune and non-immune cells (11). Other toxicities associated with CAR T cell treatment are the immune effector cell–associated neurotoxicity syndrome (ICANS), hematological toxicity due to the lymphodepleting chemotherapy, increased risk of infection due to lymphodepletion or B cell aplasia (following anti-CD19 CAR T cell administration) and macrophage activation syndrome (12). To avoid CRS and other toxicities, CAR T cells with adaptive expression systems have been developed: (i) passive control using mRNA-encoded CARs, allowing for transient CAR expression, (ii) inducible control using inducible suicide systems (13), and (iii) autonomous control *via* logic-gated CAR T cells (14).

Up to now, most of published clinical trials testing CAR T cells have used autologous T cells, i.e., cells derived from the patient for whom the product is being made. However, therapies based on autologous T cells are endowed with limitations, mainly related to the fact that the product has to be generated from each patient's cells, in a time-consuming and costly process, and with the risk of manufacturing failure (15). Indeed, delay in treatment availability can be particularly problematic in patients with highly proliferative diseases (16). An additional hurdle lies in the quantity and quality of the starting autologous T cells as patients usually receive lymphodepleting chemotherapy and/or radiotherapy (17). In addition, heterogeneity of tumor antigen expression and immune evasion mechanisms developed by tumor cells require using CAR T cell products able to target multiple antigen specificities (18). As mentioned, the amount of functional autologous T cells available in heavily pre-treated patients are often limited. In contrast, using T cells obtained from healthy donors (allogeneic T cells), provides high amounts of fully functional cells and allows to generate multiple “off-the-shelf” CAR T cell products (15, 16).

Despite many desirable traits, allogeneic CAR T cells also come with challenges. Indeed, allogeneic T cells might cause severe graft-vs.-host disease (GVHD) and the host immune system might in turn induce allorejection, which will impede anti-tumor activity. There are different ways to avoid GVHD when designing allogeneic CAR T cells, the most widely used strategy being the generation of TCR-deficient T cells using genome editing tools such as Zinc finger nucleases (ZFN) (19–21), transcription activator-like effector nucleases (TALEN) (22–24) and CRISPR/Cas9 (25–27). Strategies to reduce allorejection are being evaluated as well, testing repeated rounds of administration (28), using chemotherapy-resistant CAR T cells allowing for prolonged or deeper lymphopenia (22, 29) or genetically eliminating key molecules governing CAR T cell immunogenicity. For the latter, an attractive method uses gene editing of MHC class I molecules by disrupting the β2-microglobulin locus (30). Creating an allogeneic T cell bank is an alternative as well and this has been used mainly with virus-specific and non-modified T cells (31–33), but also with anti-CD123 retrovirally transduced CAR for the treatment of acute myeloid leukemia (34).

Glioblastoma (GBM) is the most frequent and aggressive type of primary malignant tumors originating in the central nervous system (CNS) (35). Incidence increases with age, but GBM also occurs in younger patients, with a different genetic profile (36). Despite aggressive therapies including surgery followed by concomitant chemo-radiotherapy with temozolomide (TMZ) and adjuvant TMZ (37), survival of GBM patients has only discreetly improved over the past decades. A recent systematic review showed a median overall survival of 20.7 months in clinical trials using tumor-treating fields and a 5-year survival only reaching 5.8% (38). During the last 20 years, identification of tumor-associated and tumor-specific GBM antigens led to the implementation of immunotherapy for GBM patients (39, 40). Outcome of clinical studies in primary and recurrent GBM using vaccines have largely been disappointing, and early clinical trials with immune checkpoint inhibitors didn't show positive results (41, 42). However, some promising results were recently obtained in phase I/II studies using multipeptide vaccines (43–46) or neoadjuvant immune checkpoint blockers (47–49). Given their success in other tumor indications, CAR T cells have been considered promising for GBM (2). Early phase clinical studies using CAR T cells to treat GBM patients showed that these were safe, but did not generate sufficient anti-tumor activity (50, 51). However, some monovalent CAR T cells showed tumor control (52–54) and a complete response was even reported (52). In this review, we will discuss the potential of using allogeneic CAR T cells for the treatment of patients with GBM.

## Allogeneic CAR T Cells

As mentioned before, allogeneic T cells present many advantages over autologous T cells. However, they also come with specific challenges that need to be overcome to reach clinical success. These include (i) an appropriate selection of the T-cell source, (ii) avoiding GVHD and (iii) abrogating host immune rejection to obtain robust *in vivo* activation and expansion (16).

### Sources of T Cells

**Figure 1:**
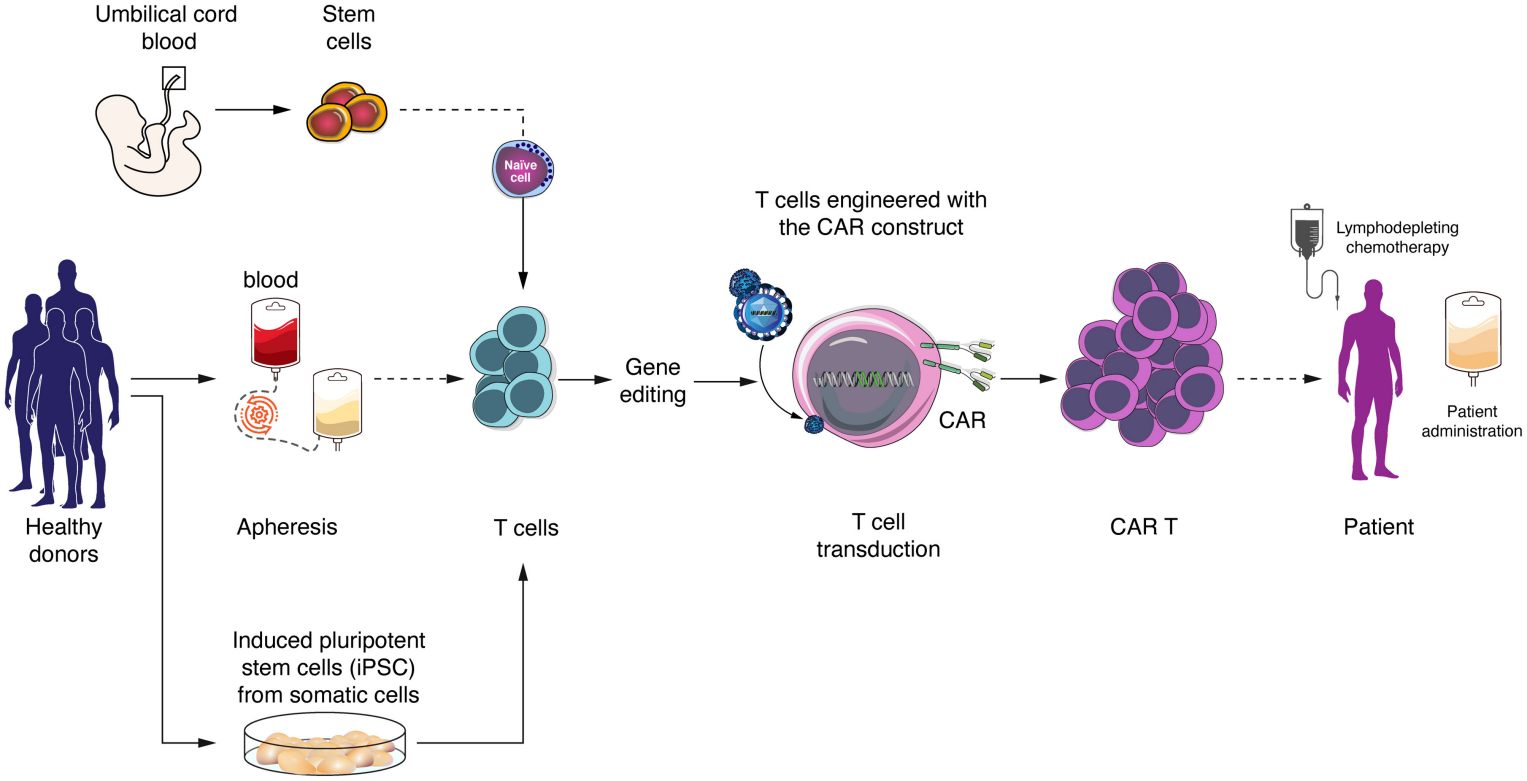
Allogeneic (“off-the-shelf”) CAR T cells generation and sources of T cells. Allogeneic T cells can be obtained from peripheral blood mononuclear cells from healthy donors, umbilical cord blood or derived from induced pluripotent stem cells (iPSCs). CAR T cells are generated by virus transduction and *in vitro* expansion before patient administration.

Patient-derived non-mobilized peripheral blood leukapheresis collection is the primary and most frequently used starting material for autologous CAR T cell manufacturing. In contrast, apheresis is performed from healthy adult volunteers in the allogeneic setting (55) (Figure 1). Using healthy donors provides high numbers of cells from a single volunteer, and peripheral blood mononuclear cells are fit, as donors, in contrast to cancer patients, do not receive chemo- or radiotherapy (16). Other cell sources can be considered for allogeneic CAR T cell development, such as umbilical cord blood (UCB)-derived T cells. GVHD frequency and intensity can be decreased when using T cells obtained from UCB, as these have reduced reactivity due to lower activation of the NF-κB pathway, resulting in decreased production of several pro-inflammatory cytokines (56, 57). In the context of hematopoietic stem cell transplantation (SCT) for treatment of hematological malignancies, UCB transplantation has indeed shown better results than matched unrelated donors and similar results as compared to matched related donor transplantation with regard to GVHD incidence, late complications and overall survival (58–60). CAR T cells derived from UCB have already been used, showing the feasibility of the approach, as well as efficacy, as UCB-derived CAR T cells were able to recognize and kill target cells (61). Another promising option is induced pluripotent stem cells (iPSCs). This allows generating pluripotent stem cells starting from adult somatic cell by introduction of specific transcription factors (62). iPSC-derived T cells have longer telomeres than mature T cells and show higher proliferation capacity. Until now, one study showed that anti-CD19 CARs can be obtained from iPSC-derived T cells, these CAR T cells being able to specifically eliminate target cells (63). However, not much progress has been reported with the use of iPSC for CAR T cell generation lately.

### Avoiding GVHD

Avoiding GVHD concentrates most efforts in allogeneic CAR T cell development, considering that GVHD is one of the main causes of death after allogeneic SCT (64). In lasts years, many groups have been working at refining the diagnostics and classification of GVHD. The current consensus defines two main categories of GVHD, acute and chronic, each divided in 2 subcategories (65). However, the literature related to CAR T cells, and especially allogeneic CAR T cells, does not address the differential impact of these therapies for each GVHD categories, particularly in chronic GVHD. As allogeneic CAR T cells advance in clinical settings, more research will be needed to understand their impact in both categories of GVHD.

Many groups consider that the principal responsible of GVHD are αβ T cells, the T cell type mostly used to generate CAR T cells (66). Two main strategies designed to reduce the risk of GVHD have been proposed, based on either selection of virus-specific T cells or genetic ablation of the TCR locus. As the risk of alloreactivity increases with donor TCR repertoire diversity and amount of T cells transferred (16), there is a rationale to use purified T cells with a low-diversity TCR repertoire. Indeed, the use of virus-specific memory T cells during hematopoietic SCT was able to control viral infections without occurrence of GVHD (67–69). However, even if repeated stimulations of donor T cells can increase virus-specific memory cells frequency, and in turn reduce the risk of GVHD, it is still not trivial to predict *a priori* the degree of alloreactivity of these cells (70). A small clinical trial using allogeneic virus-specific T cells expressing the anti-CD19 CAR construct demonstrated that these were safe and capable of anti-tumor activity without clinical manifestation of GVHD (71). New clinical trials are ongoing using anti-CD19 and anti-CD30 CAR T cells engineered with Epstein–Barr Virus-specific allogeneic T cells (72). The use of virus-specific T cells as a source of allogeneic CAR T cells remain an interesting option that needs to be fully validated for the next generation of clinical trials.

**Figure 2:**
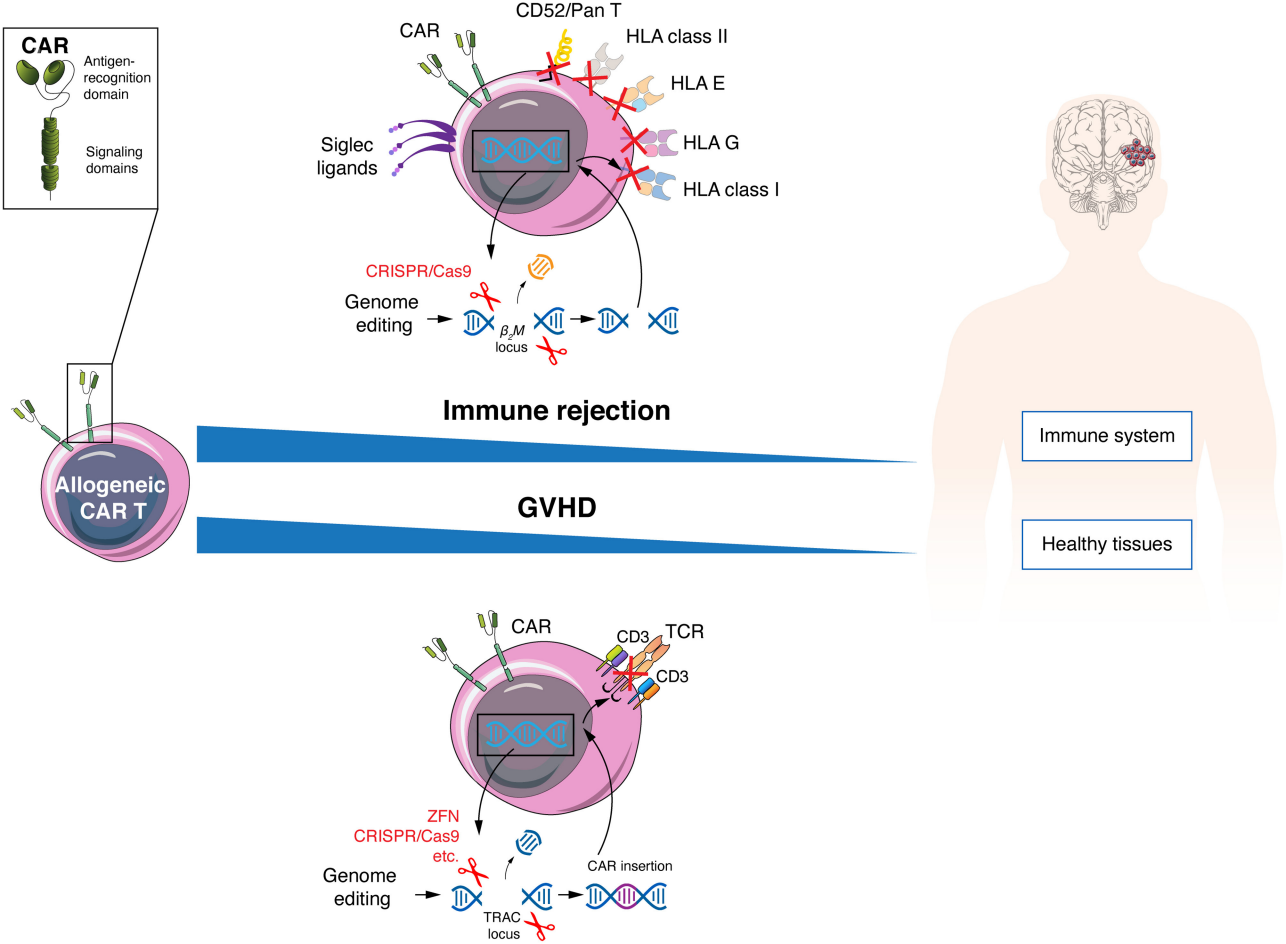
Allogeneic CAR T cells must avoid host immune rejection and GVHD. Allogeneic CAR T cells can evade the patient immune response by genetic disruption of HLA class I and II molecules, resist lymphodepleting regimens using anti-CD52 antibodies by elimination of the CD52 molecule and inhibit NK elimination by increasing expression of Siglec ligands of HLA-E and G variants. To protect patients from GVHD, allogeneic CAR T cells can be engineered to lose TCR expression.

In recent years, rapid development of gene editing technologies has provided the necessary tools to abrogate expression of endogenous TCRs in order to minimize the risk of GVHD (Figure 2). Different groups are eliminating the expression of αβ TCRs on the T cell surface through genetic knockout of exons of the TCRα constant (TRAC) and/or TCRβ constant 1 (TRBC1) or 2 (TRBC2) loci, using small interfering RNA (73), ZFN (19, 20), TALEN (24, 74), megaTAL nucleases (75), engineered homing endonucleases (76), or CRISPR/Cas9 (26, 27). In a direct comparison between TALENs, CRISPR/Cas9 and megaTAL nucleases, the latter 2 were best at TCR disruption (75). Since there is only one gene for the α-chain constant region, this seems to be the most direct and efficient approach to disrupt the αβ TCR, and is consequently the most frequently used (75, 77). Additionally, multiplex editing is possible to further modify CAR T cells. Indeed, CRISPR/Cas9 has been used to generate TCR and MHC class I deficient allogeneic CAR T cells with additional PD1 (25), Fas or PD1/CTLA4 (78) knockout. The edition of multiple genes can contribute to reduction of CAR T cell alloreactivity while improving resistance to apoptosis and immunosuppression. However, it also increases the risk of off-target cleavage that could potentially lead to an excessive proliferation of CAR T cells due to disruption of tumor suppressor genes (79, 80). One of the most interesting alternatives to gain functional advantages and avoid GVHD in a more controlled way is to introduce the CAR transgene directly into the TRAC locus. Indeed, in addition to reducing GVHD, this manipulation allows for an homogenous and regulated expression of the CAR under the control of the TCR promoter, a feature which was shown to lead to decreased CAR T cell differentiation and exhaustion (26, 76, 77, 81). This variant is also explored in field of TCR-engineered T cells with similar benefits (82, 83).

Other strategies that have been considered to avoid GVHD are the use of non-αβ T cells (84, 85) or T cells derived from a hematopoietic SCT donor. The first include a population of innate-like lymphocytes such as NK (86, 87), invariant NKT (iNKT) (88, 89), or γδ T (90, 91) cells. In the case of γδ T cells, these rare cells (5% of T lymphocytes), are able to expand *ex vivo*, show strong cytotoxic anti-tumor activity and recognize their targets independently of MHC restriction, and there are unlikely to trigger GVHD (92). Some encouraging results have been showed in pre-clinical experiments with CAR γδ T cells, including targets associated with gliomas as the disialoganglioside GD2 (90). We will discuss more on NK and iNKT cells later. Using T cells derived from an SCT donor is limited to patients who have relapsed after an allogeneic hematopoietic SCT. Here, it is possible to use the same donor-derived CAR T cells at relapse, a procedure that showed GVHD only in 6.9% of patients from a meta-analysis of seven studies (93).

### Limiting Allorejection

A second major challenge of allogeneic CAR T cell therapy is that allogeneic CAR T cells have to persist and expand *in vivo*, a feature that has been associated with response to treatment in autologous CAR T cell trials in hematologic malignancies (94, 95) and neuroblastoma (96). As commented above, allogeneic CAR T cells do not share the limitation of autologous products in T cell functionality, and the main concern to increase *in vivo* persistence is thus to reduce their immunogenicity.

The fact that allogeneic CAR T cells can be produced in greater numbers as compared to autologous CAR T cells allows for repeated administrations. Some early results using this approach in an attempt to circumvent *in vivo* rejection showed that it was feasible (97). However, repeated administration requires repeated patient immunosuppression, and repeated encounters with host immune cells increase the risk of alloreaction, at least by the antibodies produced upon previous transfusions. Aiming at a more prolonged lymphopenia is an alternative, but will require generating CAR T cells that can resist lymphodepleting agents. To do this, αβ TCR-deficient CAR T cells were made resistant to multiple purine nucleotide analogs *via* deletion of the deoxycytidine kinase gene and were shown to be capable of efficient tumor cell killing in the presence of lymphodepleting agents (29). Alternatively, CAR T cells were made resistant to depletion *via* the anti-CD52 monoclonal antibody (alemtuzumab) used as pre-conditioning regimen by knocking out CD52 (22).

Independently of the number of doses infused and of the intensity of lymphodepletion, reducing the immunogenicity of allogeneic CAR T cells is always desired, and one direct approach is the genetic abrogation of MHC class I molecules (Figure 2). Despite being highly polymorphic molecules, all share the β2-microglobulin protein, and disrupting this subunit allows the elimination of all MHC class I molecules at the T cell surface (98, 99). A second level of allorejection could be mediated *via* the presence of HLA class II molecules on CAR T cells. Indeed, activated human T cells express the MHC class II molecules DR, DQ, and DP at the cell surface, which is regulated by the class II MHC transactivator (CIITA). The function of MHC class II molecules on T cells remains controversial (100), but it is conceivable that it can induce allorejection *via* CD4^+^ T cell recognition. This issue probably could be avoided by genetic editing of the transcription factors regulatory factor X and CIITA (101, 102). Allogeneic anti-CD19 CAR T cells triple-knockout for HLA class I (β2-microglobulin KO), class II (CIITA KO) and TCR (α-chain KO) showed better persistence than double-knockout (β2-microglobulin and TCR) cells in a mouse tumor model, with anti-tumor activity, but without GVHD (103). Other cells potentially mediating an allogeneic response are NK cells (104), even if NK were shown to be functionally impaired in some tumors, particularly from hematological origin (105, 106). Expression or overexpression of inhibitory ligands could be a possible solution to prevent NK cell-mediated allorejection, with HLA-E or G (107–109) or Siglec 7/9 ligands (110, 111) being among most promising options. Finally, new alternatives are being developed to avoid CAR T cell rejection, one promising strategy being the recent generation of a CAR that mediates deletion of activated host T and NK cells through expression of an extracellular 4-1BB ligand combined with the intracellular CD3ζ signaling molecule (112).

### Addressing Tumor Heterogeneity With Modular CAR T Cells

**Figure 3:**
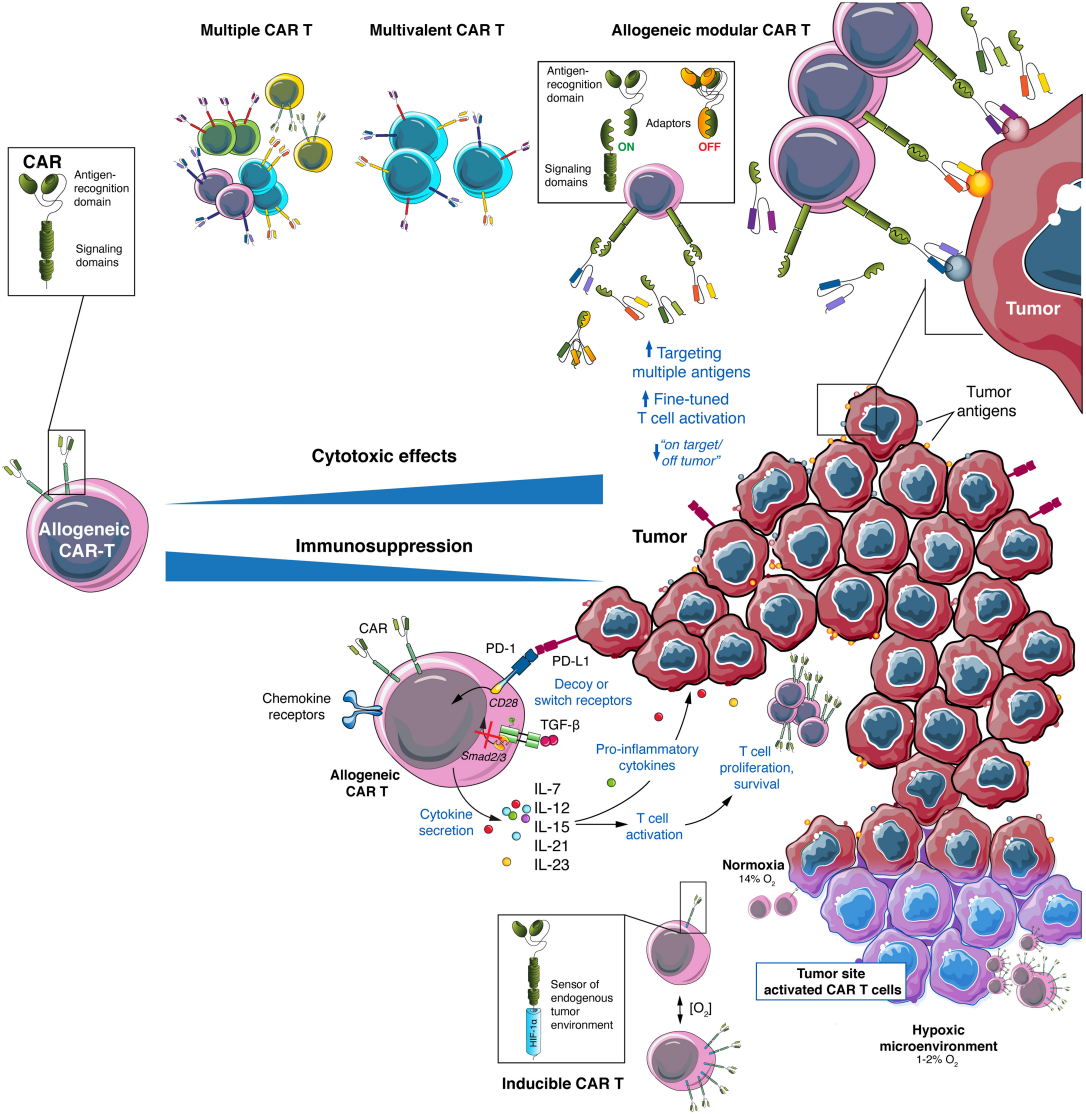
Allogeneic CAR T cells provide a versatile platform to attack GBM and its environment. GBM heterogeneity require a multitarget approach that can be achieved using allogeneic CAR T cells using multiple CAR T cell mixes, multivalent CAR T cells or by modular CAR T combined with several adaptors. To overcome the immunosuppressive TME of GBM several strategies to engineer allogeneic CAR T cells can be used: secretion of pro-inflammatory cytokines (such as IL-7, IL-12, IL-15, IL-21, or IL-23), expression of decoy or switch receptors (to change immunosuppressive signals into activating ones), expression of chemokine receptors (to direct CAR T cells to the tumor site) and generation of locally activated CAR T cells (such as hypoxia-inducible CAR T cells).

Antigen loss is a common tumor resistance mechanism to CAR T cell therapy (113) and has been reported as one of main causes of relapse in hematological malignancies (114) and GBM (52) as well as in pre-clinical models of solid cancers (115). An interesting approach to overcome antigen escape is the use of “universal” modular CAR designs. In these, the scFv targeting the antigen of interest is fused to an intermediate soluble molecule (or adaptor) which can be bound by the construct containing the activation signals expressed by the T cell (Figure 3). These CARs are based on antibody Fc receptors, streptavidin-biotin interaction, scFvs directed against a specific tag or other combinations (116, 117). Two of the most famous universal modular CARs are split, universal, and programmable (SUPRA) CARs and universal CARs (UniCAR). SUPRA CARs consist of a receptor with a leucine zipper on T cells and a separate scFv with a leucine zipper adaptor molecule targeting specific antigens (118). The design of SUPRA CARs confers some advantages, potentially significant in a clinical setting, over a classical CAR design, such as ability to change targets at will, adjusted control of activity and toxicity, and flexibility to change and combine signaling domains and immune cell types (15). The UniCAR system consists of two components as well, one being the CAR T cell that expresses a CAR directed to the nuclear antigen La-SS/B-derived peptide E5B9 and the second, termed target module, consisting of the E5B9 peptide fused to a tumor-specific antigen binding domain, typically an scFv (119). The UniCAR system can also target more than one antigen, combine different signaling domains and provide an on/off switch system that allows for a better control of CAR T cells activation (119–121).

In general, using adaptor molecules allows regulating CAR T cell activity through target selection, one or more of these targets being tackled simultaneously or sequentially. In addition, the effector activity can be turned on or off against each target separately by adding or removing the soluble adaptor, without the need to deplete CAR T cells. Thus, universal modular CAR T cells offer the opportunity to target multiple tumor antigens, with reduced toxicity. In addition to these desirable features, potential side effects of modular CAR T cells could be mitigated by a customization of the adaptor dose (122, 123). However, modular CAR T cells still bear some disadvantages related to the exogenous nature of the adaptor molecules that can generate neutralizing antibodies in the host. Additionally, each new adaptor may require its own manufacturing development, clinical validation and regulatory approval related to safety and effectiveness (124). Universal off-the-shelf and universal modular CAR could be conjugated to obtain a “fully universal” CAR that would be readily available to switch target specificity and allow fine-tuned control at the same time. This approach could be particularly suited for GBM, a highly heterogeneous solid tumor.

### Alternative Sources of Allogeneic CAR Cells: NK and NKT Cells

Besides T cells, other cells can be used to generate CARs. NK cells are the most explored alternative to T cells, due to their potent cytotoxic anti-tumor activity combined with a favorable safety profile. A smaller risk of inducing GVHD has been historically associated with adoptive transfer of allogeneic NK cells because, as opposed to T cells, NK cells kill independently of MHC expression (125). In addition, allogeneic CAR NK cells do not require genetic modifications such as TCR deletion, which makes it easier to obtain cellular product devoid of any risk of GVHD (126). Even more interesting is the differences in the cytokine profile of NK cells compared to T cells, as NK cells do not produce IL-1 and IL-6, the main cytokines involved in CRS (127). Despite the fact that macrophages are usually the main source of cytokines such as IL-6, there is a debate about the role of activated T cells in IL-6 secretion during CAR T cell therapy, some studies pointing to T cells as the main source of IL-6 (128, 129), while others showing no IL-6 production by CAR T cells (130). However, generating CAR NK cells faces several practical issues, from the lower amount of these cells in blood of adult donors compared to T cells, to a limited ability to expand *ex vivo*, although progress is being made in the latter through the use of improved protocols with different cytokines such as IL-15 and IL-18 (131, 132). In addition, genetic engineering of NK cells is less efficient than with T cells, with low transduction rate and loss of cytotoxic activity (126, 133). However, CD19 CAR-transduced NK cells from cord blood were recently shown to induce 64% of complete responses in patients with hematological malignancies, with favorable cell product attributes, i.e., CAR expression, engraftment and expansion *in vivo*, and without showing serious toxicity (134). In addition, given the existence of various NK cell subsets, important differences in phenotype and functional activity can be observed depending on the cells used (135, 136). NK cell lines are an alternative with potential to overcome most primary NK cells handicaps. These cells lines are rare but at least one of them, NK-92, is endowed with a high cytotoxic activity due to expression of many NK activating receptors and loss of some of the NK inhibitory receptors (137, 138). Advantages of NK-92 cells are their unlimited expansion *in vitro* and easy maintenance in culture (requiring only IL-2 supplementation). In addition, they are an homogeneous source of NK cells with an invariant phenotype and cytotoxic activity, a safe profile, and are easily modifiable through genetic modifications (133, 137, 139). Additionally, using CRISPR/Cas9 editing, NK-92 cells have been modified to re-express CD16 or DNAM-1, allowing to increase their anti-tumor cytotoxic activity to levels close to primary NK cells (138). As NK-92 cells originate from a human NK cell lymphoma, one limitation as cell therapy is the mandatory irradiation of these cells before infusion in patients to avoid any risk of malignant expansion. Irradiation does not affect functionality of NK-92 cells, including cytotoxic activity, but impairs proliferation *in vivo*, reducing NK-92 engraftment to a few days (126, 139). In order to overcome this limitation, NK-92 CARs must be infused several times in patients, a concept that has already been proven as feasible in an anti-Her2 therapy (140–142). Furthermore, despite the fact that CAR NK-92 cells showed pre-clinical activity against several tumor targets such as CD19, EGFR, Her2, and PSMA, they still need to be validated in a clinical setting (137, 143). Finally, among other population that have been explored to generate allogeneic CARs are iNKT cells (144). iNKTs combine a strong cytotoxic capacity with an activation restricted to the monomorphic CD1d molecule (145), reducing the risks of off-target toxicity, since CD1d is expressed mainly by antigen presenting cells such as dendritic cells and B cells (146). Interestingly, allogeneic iNKT cells have been associated with a protective effect against GVHD (147, 148). Similar to NK cells, iNKT cells are more difficult to culture, transform and expand that T cells and repeated cell administration or adjuvant use of IL-2 would be necessary to achieve persistence and anti-tumor efficacy (144, 149). However, several groups are working to improve iNKT cell culture and expansion protocols, with recent advances potentially making this population an efficient and flexible platform for next-generation CAR therapies (88, 150).

## CAR T Cells for Brain Tumors

### Challenges Associated With Tumor Location in the Brain

Although solid tumors are challenging for CAR T cell therapy, GBM are endowed with specific hurdles. First, entrance of immune cells, including CAR T cells, into the CNS is usually low, since migration of these cells into the CNS is limited by the endothelial blood–brain barrier (BBB) and the epithelial blood–cerebrospinal fluid barrier (151). Second, high inter- and intratumoral heterogeneity is one of the hallmarks of GBM, making selection of tumor antigens for CAR T cell design more challenging. Finally, GBM displays an immunosuppressive environment induced by the tumor itself, by recruited immune cells and by standard radiochemotherapy treatments that hamper CAR T cell activity (152). Finally, there is a great limitation of relevant models in GBM to assess CAR T cell function, study resistant phenotypes and test combinatorial strategies (152).

### Making CAR T Cells Reach the Tumor in the Brain

The first challenge of CAR T cell therapies against GBM is to achieve active trafficking of the effector CAR T cells to the tumor site (153). In the published clinical trials, GBM patients were treated with anti-IL13Rα2, Her2, or EGFRvIII CAR T cells, and investigators explored both local and systemic administration routes. The main achievement of these trials was to provide evidence of safety of anti-GBM CAR T cells and potential for clinical efficacy, with some durable responses reported (50, 51). However, the majority of patients treated did not experience clinical benefit. A critical analysis of the main factors contributing to the low efficacy of these trials pointed to the necessity to improve CAR T cell trafficking and engraftment (50). Relevance of the administration route was evidenced with one of the more remarkable results showing tumor regression in a patient receiving multiple infusions of IL13Rα2-specific CAR T cells. The patient was treated CAR T in a sequential manner, using first the intracavitary and then the intraventricular route. While the first treatment resulted in local tumor recurrence, intraventricular infusion caused regression of all CNS lesions (52). This correlates with pre-clinical brain tumors studies, in which evidence points to an increased anti-tumor activity after locoregional administration of CAR T cells (154–156).

Another way to enhance trafficking to the brain would be to engineer CAR T cells with specific chemokine receptors that improve infiltration into the tumor (157). As an example, anti-GBM CAR T cells targeting CD70 showed increased trafficking to the tumor site and a better anti-tumor activity after being transduced with CXCR1 or CXCR2 (158). CXCR1 and CXCR2 are receptors for IL-8 (CXCL8), an inhibitory chemokine involved in recruitment of tumor-associated neutrophils or myeloid derived suppressor cells (MDSCs) (159), tumor proliferation and angiogenesis (160). Furthermore, despite the fact that most of chemokines attracting T cells are downregulated in GBM, some are upregulated. Two examples are CCL17 and CCL22, two chemokines involved in T regulatory (Treg) recruitment (161), that can be used to attract CAR T cells if the latter are transduced with their cognate receptor, the CCR4 molecule. Another argument to justify transfection of CAR T cells with CCR4, which also holds true for CCR2, is based on their ability to bind to CCL2, a chemokine expressed in gliomas that has been demonstrated to recruit T cells to tumor site *in vivo* (162). Finally, other approaches point to overexpressing some of the chemokine receptors involved in T cell trafficking to inflammatory sites, such as CXCR3, owing to expression of its ligands, CXCL9 and CXCL10, in the GBM tumor microenvironment (TME) (163). Indeed, CXCR3 signaling through interaction with CXCL9 and CXCL10 has been shown to play a relevant role in tumor homing of effector T cells (164).

### *In vivo* Monitoring of Cellular Products and Treatment Efficacy

Monitoring persistence and functionality of CAR T cells is essential to improve effectiveness of anti-tumor therapy. In contrast to hematological malignancies, monitoring CAR T cells at the tumor site in solid tumors is usually more difficult and new strategies to follow and evaluate CAR T cells in the tumor and/or in periphery must be designed. Conventional brain imaging using computer tomography (CT) or magnetic resonance imaging (MRI) with contrast are commonly used for brain tumor diagnostic and disease follow-up. One of the issues associated with the use of MRI in patients treated with immunotherapy is a phenomenon known as pseudoprogression, i.e., increase of lesion sizes related to treatment, which simulates progressive disease (165). Pseudoprogression, which has been associated with favorable prognosis in some instances (166), is difficult to distinguish from true tumor progression. This was evidenced by a phase I dose-escalation trial with an anti-Her2 CAR. In the weeks following CAR T cell infusion, several patients showed a progression-like image with increase in peritumoral edema, but all survived more than 6 months, suggesting pseudoprogression and not true tumor progression (53). New imaging-based methods are therefore needed to more accurately follow CAR T cell expansion at the tumor site.

The *in vivo* detection of cell therapy products through ^19^F-based MRI after endocytosis of ^19^F-dense perfluorocarbon nanoemulsions, still in early development, is a promising option to monitor CAR T cell infiltration and survival in the tumor during clinical trials (167). Based on the high *in vivo* sensitivity of nuclear imaging methods, one interesting variant to ^19^F MRI cell detection are radionuclide-based imaging methods, mainly positron emission tomography (PET) and single photon emission computed tomography (SPECT) (168–170). Due to high sensitivity of PET and SPECT, higher than that of MRI techniques, much lower concentrations of radiolabeled compounds can be used, making possible to avoid interference with cell function and viability (168, 171). Using a ^89^Zr-labeled anti-ICOS antibody, it was shown that PET can be a useful tool for *in vivo* tracking of CAR T cells in an orthotopic murine tumor model of lymphoma (172). One disadvantage of PET and SPECT being that they have a limited spatial resolution, combination with CT provides a first approach to surpass this limitation (173). A first successful use of PET/CT to detect tumor-infiltrating CAR T cells was reported in a mouse model of an anti-GD2 CAR T cells, the vector used co-expressing a dihydrofolate reductase enzyme that generates an ^18^F-probe for PET (170). However, since PET-CT has disadvantages as well, such as errors during the co-registration of images and high radiation doses due to CT scans, approaches that combines nuclear imaging techniques with MRI are being developed (173, 174). PET-MRI and SPECT-MRI combine the high sensibility to visualize physiological process with the capacity to show anatomical structures and are already becoming one of most powerful imaging platforms to study CAR T cells (174). On the other hand, use of perfusion-weighted imaging approaches in the MRI field, both dynamic contrast enhanced and dynamic susceptibility contrast-enhanced seems to be able to differentiate between the effects of chemotherapy and radiotherapy on tumor progression in high-grade gliomas (175). Both methods have shown a relatively good accuracy in individual studies, but further investigation and standardization is needed until they can be used for CAR clinical trials (175). Another promising alternative, limited to patients with surgical re-interventions after CAR T cell treatment, is the *in-situ* analysis of CAR T cells. This uniquely allows to analyze the amount and phenotypical characteristic of CAR T cells that can successfully migrate to the tumor and evaluate, as a possible prognosis of tumor evasion, the changes in CAR T cells antigen expression after treatment (54). In addition to MRI, another way to monitor tumor evolution under CAR T cell therapy is the detection of target antigen expression, using qPCR or immunohistochemistry on samples obtained from the tumor site (152). However, in anti-CD19 CAR T cell pre-clinical experiments, a decrease in surface CD19 expression was reported without a significant decrease in mRNA levels, leading to a debate about the reliability of qPCR measurements as surrogate of therapy efficacy against an specific antigen (176).

Although direct analysis of tumor evolution and detection of CAR T cells at the tumor site should ideally provide a direct correlation with functionality, CAR T cell assessment in the peripheral blood has some advantages as compared to reoperation or *in situ* measurements, being less invasive and safer. In this direction, analysis of the number of CAR T cells, absolute or in proportion to overall T cell populations, associated with expression of functional and exhaustion markers, shall provide an accurate picture of therapy efficacy. However, since, in GBM, intracranial administration CAR T is preferably used, it is necessary to know whether CAR T cells can reach and be detected in the periphery. A first report showed that CAR T cells administered intraventricularly, and, in lesser amounts, intratumorally, can reach the periphery, allowing to use flow cytometry to measure CAR T cells in that compartment (155). A recently developed approach makes use of liquid biopsies, which is a non-invasive technique that can be used to monitor CAR T cell persistence and tumor progression through analysis of circulating tumor (ct) or CAR DNA (177). Although initial reports showed that gliomas had lower levels of ctDNA compared to others tumors (178), recent reports pointed to the use of ctDNA or circulating cell-free DNA as a non-invasive measure of response to therapy in brain tumors (179, 180). Another recently developed platform is the detection of tumor mitochondrial DNA (tmtDNA), a technique that showed 3 times better detection rates as compared to ctDNA, and can be used in cerebrospinal fluid (CSF). tmtDNA appears to be a more sensitive method to analyze tumor change following CAR T cell treatment, and can allow overcoming limitations that other methods have shown in GBM (181). It is important to note that the anatomical structure of brain, especially the BBB, makes CSF a relevant fluid that might provide a more accurate picture of tumor treatment efficacy than plasma, including the measure of certain biomarkers (182).

### CAR T Addressing GBM Heterogeneity

Engineering successful CAR T cell therapies against GBM has two major prerequisites: (i) the choice of antigen, the goal being to target the high molecular heterogeneity inherent to GBM, and (ii) the modulation of the immunosuppressive TME to allow CAR T cell function. Analysis of clinical trials with CAR T cells for solid tumors suggests that improvements in cancer cell recognition as well as in CAR T cell persistence and activity, especially in an immunosuppressive TME, will lead to increased efficacy (183, 184). CARs that target multiple tumor antigens might allow enhancing tumor cell detection; however, increasing the number of antigens also increases the risk of “on-target off-tumor” effects that may cause serious damage to healthy tissues. Such toxicity has been observed with anti-CD19 CAR T cells, affecting dispensable B cells (185) but also brain mural cells, leading to neurotoxicity (186).

As many solid tumors, GBM displays a high intratumoral heterogeneity, with different tumor cell clusters showing differences at the genotype level (187). Single-cell transcriptomic showed that several cell types coexist in the same GBM sample, with a high degree of plasticity between the different states (188). Challenges in addressing GBM heterogeneity also lie in the fact that a small subset of tumor cells can actively sustain heterogeneity (189, 190) and that a relative small population of cancer stem cells is responsible for tumor recurrence (191, 192).

In addition to the need to target all cells within a tumor cell population, addressing antigen loss, which is a common risk when a single antigen is targeted, is required (193–195). In an attempt to predict efficacy of CAR T cells targeting two or three GBM antigens, one study applied a binominal mathematical model, using expression of three of the well-described GBM-associated antigens, Her2, IL-13Rα2, and EphA2. In primary GBM samples, the model predicted that targeting two of the three antigens would result in higher efficacy as compared to single antigen targeting, but that addition of the third antigen would not improve the outcome (193). Interestingly, in mouse models, authors compared two alternative strategies, one with a bispecific CAR and the other with a 1:1 pool of monospecific Her2- and IL-13Rα2-specific CAR T cells. Bispecific CAR T cells demonstrated a better anti-tumor activity and higher *in vitro* activation, with increased IFN-γ and IL-2 secretion, and improved cytolytic activity (193). These results encourage the development of a dual, both bispecific or tandem design CAR T cell therapy against Her2 and IL-13Rα2 in GBM (193, 194). Similar approaches to avoid antigen escape have been tested in breast cancer with CAR T cells targeting Her2 and MUC1 (196) and in B-ALL with CAR T cells targeting CD19 and CD22 (197). However, even if targeting two antigens can decrease the probability of tumor escape, this may not be enough in the case of highly heterogeneous tumors to reach complete remission (114). To enable targeting a wider proportion of GBM patients, a trivalent CAR targeting Her2, IL3Rα2, and EphA2 was generated. Trivalent CAR T cells displayed increased IFN-γ and IL-2 secretion after tumor recognition compared to monovalent and bivalent constructs, and the trivalent CAR therapy was able to eliminate nearly all tumor cells in an orthotopic patient-derived xenograft (PDX) mouse model (198).

In view of these results, developing a pool of CAR T cells with different antigen specificities would result in a flexible platform that could be adjusted to the antigen profile of each patient in terms of antigen expression. Using allogeneic cells would enable the generation of CAR T cells specific for antigens of choice or even a bank of “à la carte” CAR T cells specific to the major antigens expressed by a given tumor type (Figure 3). As mentioned above, different strategies are available to design CAR T cell therapies targeting multiple antigens, such as co-administration of two or more CAR T cell populations, each bearing a different antigen specificity, or the simultaneous expression of two, potentially three, CAR molecules in the same T cells, such as with bispecific and tandem CARs (113). In this regard, allogeneic CAR T cells is a promising option for multi-targeting approaches. Whereas, a limited number of autologous T cells is usually available, allogeneic T cells can be obtained in high numbers, allowing manufacturing different CAR T cells populations. In addition, it allows compensating for the lower efficiency of transduction with viral vectors with higher packaging capacity, when a strategy using multiple CAR molecules is preferred. In addition, availability of multi-antigen targeting CAR T cells at the time of patient diagnostic will allow rapid administration and prevent the risk of manufacturing failure. Other options to target multiple antigens are under development and include the combination of CAR T cells and bispecific T cell engagers (BiTE), in the so-called CART.BiTE strategy (199). As an example, heterogeneous GBM were eradicated in mouse models using a combination of EGFRvIII-directed CAR T cells and a secreted BiTE targeting wild-type EGFR (200).

### CAR T Cells That Modify the TME

The GBM immunosuppressive TME has been regarded as one of the main obstacles to a successful CAR T cell therapy (51). GBM contains tumor and non-tumor cells that generate a hostile environment dampening T cell function and survival (201–203). First, at the cellular level, GBM cells are able to recruit and polarize immune cells to a regulator phenotype, the better described examples being Tregs, tumor-associated M2 macrophages and MDSCs (203, 204). Second, at the molecular level, cells in the GBM TME are able to express inhibitory ligands such as PD-L1, CD95-L, or non-classical MHC class-I proteins (205) and to secrete immunosuppressive cytokines such as TGF-β and IL-10 (206). Finally, at the metabolic level, TME cells are able to decrease relevant metabolites such as glucose *via* increased consumption, or to deplete relevant amino acids, such as tryptophan, *via* IDO1 (indoleamine 2,3-dioxygenase 1) secretion, limiting T cell function. These phenomena are in addition favored in hypoxic conditions, which is common in GBM (203, 207).

#### Reshaping Immune Cells in the TME

One strategy to target immunosuppression at the TME of solid tumors using CAR T cells is based on the secretion of pro-inflammatory cytokines at the tumor site, with the aim to reprogram infiltrating immune cells while enhancing CAR T cell killing function. Cytokines tested include IL-7 (208–210), IL-12 (211–213) and IL-18 (214, 215). In GBM, pre-clinical data showed synergy of CAR T cells co-expressing IL-15 (195), IL-12, and IL-18 (216). IL-21 is another cytokine that is being considered due to its role in TME modulation (217). In fact, CAR T cells cultured *in vitro* with IL-21 showed a higher efficiency in controlling *in vivo* tumors (218). In addition, a pre-clinical study comparing CAR T cells secreting different γ-chain-cytokines (IL-2, IL-7, IL-15, and IL-21) found increased anti-tumor activity with all the cytokines tested, but effects were mediated through different mechanisms (208). Recently, transfecting CAR T cells with the IL-12β p40 subunit allowed production of IL-23 after activation, which led to an increase in cell proliferation and survival. This strategy resulted in enhanced anti-tumor activity, due to autocrine IL-23 signaling, in xenograft and syngeneic mouse models (219). A frequently reported CAR T cell design used to deliver cytokines and modulate the immunological balance at TME are TRUCKs (“T cell redirected for universal cytokine-mediated killing”). TRUCKs incorporate an NFAT (nuclear factor of activated T cells)-mediated signaling getting activated upon CAR engagement of its cognate antigen and leading to local secretion of a cytokine of interest (220). One limitation of the original TRUCKs was the necessity of transducing two vector constructs, but recently a modular “all-in-one” vector system was developed, resulting in enhanced cytotoxic activity and safety (216).

The increasing amount of results with CAR T cells secreting different cytokines suggest that selecting the appropriate cytokine could be a challenge and that, in some cases, combinations might be a better option. Since most cytokines are pleiotropic and can induce both immunostimulation or immunosuppression according to the immune contexture, their use is still in debate and more studies are needed to optimize their combination (183, 221). Also, as high levels of cytokine secretion is associated with two main CAR T cells toxicities—CRS and ICANS—the possible toxicity of additional cytokines, as we mention before for reshape TME, must be careful monitoring. Cytokines as IL-15 are strongly associated with ICANS, but direct neutralization of IL-15 or other pro-inflammatory cytokines must impair T cell activity, then alternative treatments directed to the inflammatory process itself could be more appropriate, including blockade of the signaling of IL-6, IL-1 or GM-CSF and the use of corticosteroids (222, 223).

Lastly, a new possibility to modify the TME is to target directly the immunosuppressive cells. CAR T cells targeting M2 macrophages are currently being developed using the most recent, and extensive, phenotypic characterization of these cells in GBM (224, 225).

#### Resisting Immune Checkpoint Inhibition

The other main strategy to target immunosuppression at the TME is to avoid immune suppression signals by blocking them or turning them into activating ones, mainly based on decoy and chimeric switch receptors. Chimeric switch receptors, also called inverted receptors, are composed of a receptor for an immune inhibitory signaling molecule as extracellular domain, and of the cytoplasmic domain of an immunostimulatory molecule as intracellular domain, allowing to turn an inhibitory signal into a stimulatory one (157). An interesting use of this design combines the extracellular portion of PD1 with the intracellular signaling domain of the costimulatory molecule CD28. CAR T cells with this PD1CD28 switch receptor and specific for CD19, mesothelin or PSCA, showed increased cytotoxicity and improved tumor control in several established solid tumor models (226). Chimeric switch receptor were also designed by combining the IL-4R extracellular domain with the intracellular signaling domains of IL-7 or IL-21, resulting in CAR T cells with enhanced anti-tumor activity against IL-4^+^ tumors (227, 228). Decoy, or neutralizing, receptors, including dominant-negative receptors, are also an approach that can be used to avoid TME immunosuppressive signals. In this case, receptors do not transmit any signal to the cell and can contribute to eliminate inhibitory cytokines from the TME. In a first attempt to use this strategy, CAR T cells were engineered to overexpress a truncated receptor for PD1, without the transmembrane and intracellular signaling domains of the natural receptor, resulting in non-transduction of the signal upon binding and conferring resistance to PD-L1/2 immunosuppression. This led to an increase in effector functions and better tumor control in pre-clinical experiments (229). A similar approach was shown to be successful when targeting TGF-β *via* introduction of a dominant-negative TGF-β receptor in an anti-PSMA CAR. This construct resulted in increased proliferation rate and higher persistence of CAR T cells in mice, combined with a less exhausted phenotype, leading to enhanced anti-tumor activity (230). A recent report used anti-CD19 CAR T cells with concomitant expression of an IL-6 neutralizing receptor that reduced CRS by eliminating soluble IL-6, without affecting anti-tumor activity (231). In an alternative approach, genetic editing tools such as CRISPR/Cas9 can be used to disrupt immunosuppressive signals in the TME (232). PD1 gene (*Pdcd1*) disruption using CRISPR/Cas9 in anti-CD133 CAR T cells was shown to increase tumor killing *in vitro* while inhibiting *in vivo* tumor growth in a orthotopic glioma xenograft model (233). In a stimulating attempt to create universal allogeneic CAR T cells against EGFRvIII, the CRISPR/Cas9 system was also used to disrupt *Pdcd1*, together with the TRAC and β2-microglobulin genes. These triple negative CAR T cells showed improved persistence and anti-tumor activity in NSG mouse models of human GBM. Interestingly, this was observed only when locoregional administration was used, suggesting the relevance for a successful anti-GBM therapy of immune checkpoint signaling blockade and local infusion of CAR T cells (234).

#### Targeting Metabolic Checkpoints

Since a hypoxic microenvironment is found in many solid tumors, and is considered a hallmark of GBM (235), some authors are considering turning it into an advantage. The best example is the development of a CAR containing sub-domains of hypoxia-inducible factor-1α to induce CAR T cell activation only under the low oxygen levels found in TME (Figure 3). These oxygen-sensitive CAR T cells showed a more tumor-localized function that enhanced efficacy, allowing the use of tumor associated antigens with lower risk of side effects (236). However, this strategy would still come with the risk of unanticipated CAR activation in regions of the human body bearing low oxygen levels, especially in patients with concomitant pathological conditions such as infections or inflammation (237).

## The Need to Have Immunocompetent Pre-clinical Models That Reflect the Complexity of the GBM Microenvironment

**Figure 4:**
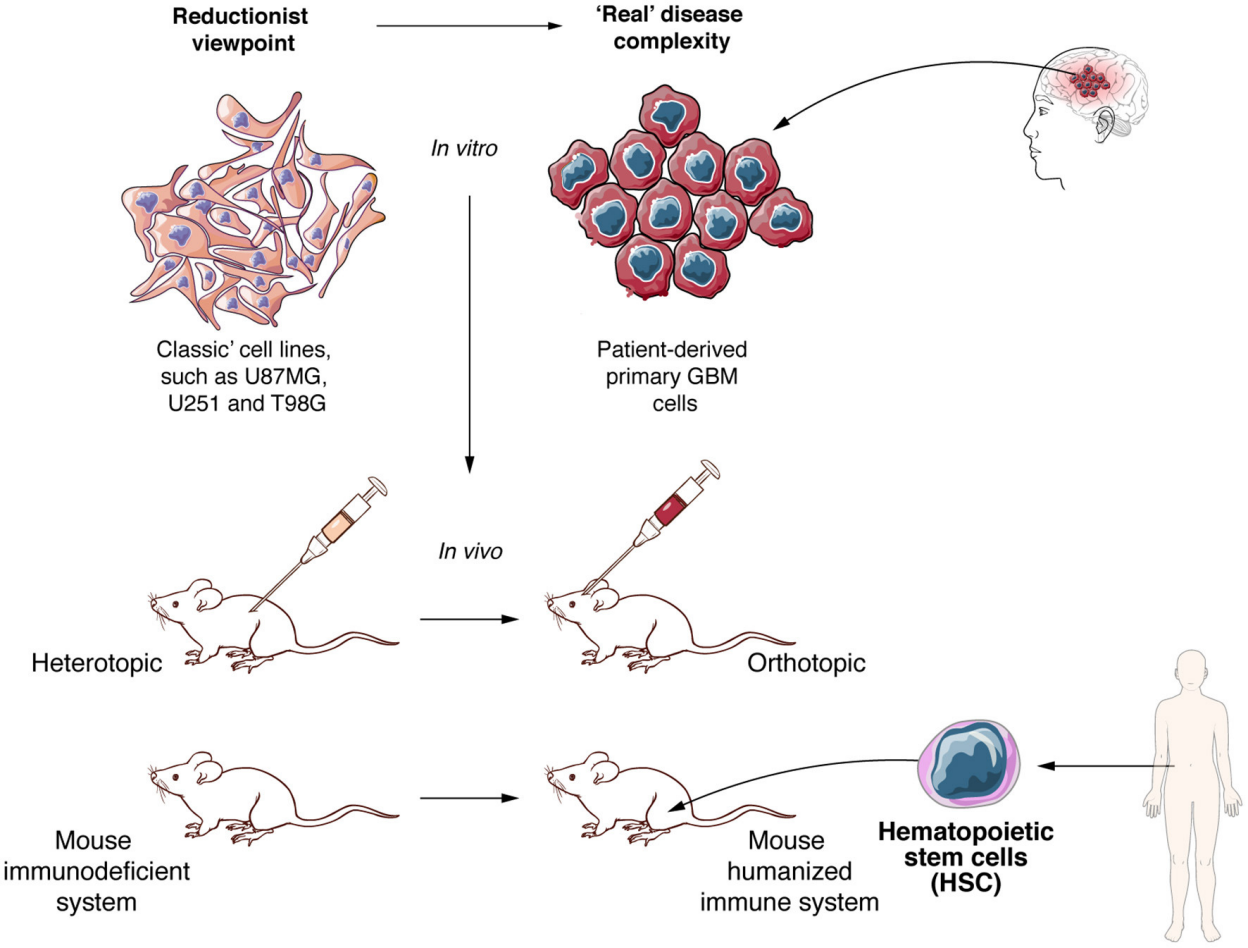
To develop new and effective anti-GBM therapies better experimental models are required. Experimental *in vitro* and *in vivo* models need to move to more realistic settings. Patient derived primary cell lines and xenograft models, orthotopic tumor growth and mice with humanized immune system must become the standard to evaluate allogeneic CAR T cells against GBM.

Like any other therapy, CAR T cell therapy needs to be tested in pre-clinical models before reaching clinical trials. Available experimental models can be categorized in two main groups, both with advantages and disadvantages. On one side, reductionist models are being used to elucidate the specific role of molecules or biological processes, and make direct measures of functional effects such as cytotoxicity without most of the complex relations present in a live organism. On the other side, “close to reality” models aim at recapitulating the tumor and its interaction with other components of the organism such as the immune system, and are more focused on evaluating the probable efficacy of therapies (238) (Figure 4). CAR T cell cytotoxic functions can be directly tested using classical human GBM cell lines, such as U87MG and U251MG, but despite the fact that these experiments can provide a proof of concept, these models suffer from several limitations. Long-term *in vitro* culture induces significant changes at the genetic and transcriptional level, including loss of stemness attributes and differentiation to an astrocytic phenotype, diverging away from the characteristics of human GBM (239). Since human CAR T cells need to be tested in xenograft models and given that GBM cells lines are not optimal to recapitulate the heterogeneity of human tumors *in vivo*, many groups have moved to alternative models such as GBM stem cells (GSC), PDX or organoids (238). GSC culture conditions induce the preferential expansion of glioma stem cells and are a useful tool to study GBM biology, including *in vivo* tumor development and heterogeneity, and may also predict efficacy of some therapies (240). Human GBM-derived PDX models provide a more precise representation of tumor heterogeneity than other available models, including the ability to reproduce many of the interactions between tumor and the TME (241, 242). Due to these characteristics, PDX models, in particular when used orthotopically, appear to more closely recapitulate the real disease (243–246). However, after several *in vivo* passages, PDX tumors can also notably diverge from the original primary tumor, both at genetic level and at subpopulation composition (247, 248). In recent years, organoids have become another promising alternative to reproduce GBM in mouse models. Combining GSCs and iPSC, several researchers were able to generate highly heterogeneous 3D cultures, integrating neoangiogenesis and a hypoxic milieu and making these a clinically relevant option to test new anti-GBM therapies (240, 242, 249).

In addition to the challenges of any xenograft GBM model to represent tumor heterogeneity, immunosuppressed mouse models are not capable of reproducing the full interaction of tumor or CAR T cells with the immune system. As such, one of the most commonly used immunocompromised models, the NOD-scid/IL2R-γ^null^ (NSG) mouse model, does not allow interrogating the effect of infiltrating immune cells (250, 251). In addition, interactions of CAR T cells with other immune or stromal cells, the potential “on-target off-tumor” effects and the generation of CRS cannot be modeled either (252). To overcome limitations in assessing CAR T cell interaction with other immune cells and simulating the CRS, important progress has been made to develop mouse models with a humanized immune system (253, 254). Through human hematopoietic CD34^+^ stem cell transplantation in immunosuppressed mice, including NSG mice, many groups were able to develop mice incorporating many cellular components of the human immune system, including both myeloid and lymphoid lineages (255, 256). The humanized mouse field is in constant progress, with new mouse models being able to resemble more accurately the human immune system. As an example, NSG or NOD/Shi-scid/IL-2Rγ^null^ (NOG) mice that express human cytokines such as G-CSF, GM-CSF, or IL-3, lead to better engraftment, generation and development of the human immune system after injection of human CD34^+^ stem cells (257, 258). Another consideration in designing relevant GBM models is the inoculation site of tumor cells. Orthotopic engraftment must be preferred, as it confers an adequate environment to GBM development and better reflects the human disease (238). Finally, other animal models could be explored, such as immunocompetent dogs that spontaneously develop high grade gliomas and show some of the features of human GBM, such as tumor heterogeneity, in presence of a functional immune system (259, 260) (Figure 4).

## Conclusions and Perspectives

CAR T cell based therapies are transforming the treatment of hematological malignancies and have the potential to do the same in solid tumors (184). However, despite showing some evidence of anti-tumor effect, CAR T cell therapies against GBM still need to prove their efficacy to become a viable and impactful therapeutic option (152). To this day, all 21 clinical trials (Supplementary Table 1, updated December 2020 in https://clinicaltrials.gov/) using CAR T against GBM are based on autologous CAR T cells. In addition to addressing the many obstacles raised by solid tumors and GBM in particular, using allogeneic T cell sources might be part of successful future strategies. Several advantages make allogeneic CAR T cells a relevant option for GBM patients (16). Allogeneic CAR T cells have the advantage of being ready to use and can be readily administered to patients from the moment of diagnosis. In addition, healthy donors provide high amounts of T cells, without loss of functionality due to exhaustion or suppression, which are commonly found in T cells derived from cancer patients (261) and particularly in GBM (262). However, using allogeneic CAR T cells comes with the need to overcome host immune rejection and to minimize GVHD (16). The first can be accomplished mainly through elimination of HLA molecules and the second by knocking-out the TCR or by using tumor site-specific activation strategies such as hypoxia-activated CAR T cells. Nonetheless, an important feature of allogeneic cells is that, as opposed to autologous T cells usually used to generate monovalent CAR T cells, allogeneic T cells can be used to generate several CAR T cell products with different antigen specificities or multivalent CAR T cells, enabling to overcome GBM heterogeneity. It could also be used as a personalized therapy to target the set of antigens expressed by a given patient. Allogeneic CAR T cells could further be combined with modular CAR strategies to make “fully universal” CAR T cells that combine availability of allogeneic cells and flexibility of modular designs. As such, allogeneic CAR T cells would not only be able to target different GBM antigens but also to target the TME, offering a new promising field that could overcome the limitations of current CAR T cell strategies.

Today, there is virtually no limit to synthetic biology and cell engineering, providing a platform to develop new therapies against GBM. Together with the identification of new and highly tumor-restricted antigens, the development of more representative experimental models and improved imaging techniques to assess tumor response and CAR T cell features *in vivo* will be part of that therapeutic challenge. In the next decade, the neuro-oncology field will most probably witness the advent of allogeneic CAR T cells, engineered immune cell products endowed with multiple specificities and resistant to host rejection, hopefully allowing transition to improved patient outcome.

## Author Contributions

All authors listed have made a substantial, direct and intellectual contribution to the work, and approved it for publication.

## Conflict of Interest

The authors declare that the research was conducted in the absence of any commercial or financial relationships that could be construed as a potential conflict of interest.

## References

[1] Jackson HJ Rafiq S Brentjens RJ. Driving CAR T-cells forward. Nat Rev Clin Oncol. (2016) 13:370–83. 10.1038/nrclinonc.2016.36 27000958 PMC5529102

[2] June CH Sadelain M. Chimeric antigen receptor therapy. N Engl J Med. (2018) 379:64–73. 10.1056/NEJMra1706169 29972754 PMC7433347

[3] Schuster SJ Svoboda J Chong EA Nasta SD Mato AR Anak Ö. Chimeric antigen receptor T cells in refractory B-cell lymphomas. N Engl J Med. (2017) 377:2545–54. 10.1056/NEJMoa1708566 29226764 PMC5788566

[4] Neelapu SS Locke FL Bartlett NL Lekakis LJ Miklos DB Jacobson CA. Axicabtagene ciloleucel CAR T-cell therapy in refractory large B-cell lymphoma. N Engl J Med. (2017) 377:2531–44. 10.1056/NEJMoa1707447 29226797 PMC5882485

[5] Park JH Rivière I Gonen M Wang X Sénéchal B Curran KJ. Long-term follow-up of CD19 CAR therapy in acute lymphoblastic leukemia. N Engl J Med. (2018) 378:449–59. 10.1056/NEJMoa1709919 29385376 PMC6637939

[6] Maude SL Laetsch TW Buechner J Rives S Boyer M Bittencourt H Bader P. Tisagenlecleucel in children and young adults with B-cell lymphoblastic leukemia. N Engl J Med. (2018) 378:439–48. 10.1056/NEJMoa1709866 29385370 PMC5996391

[7] fda.gov. U.S. Food & Drug Administration. FDA Approves Axicabtagene Ciloleucel for Large B- Cell Lymphoma. (2017). Available online at: https://www.fda.gov/drugs/resources-information-approved-drugs/fda-approves-axicabtagene-ciloleucel-large-b-cell-~lymphoma (accessed April 10, 2020).

[8] fda.gov. U.S. Food & Drug Administration. FDA Approves Tisagenlecleucel for B- Cell ALL and Tocilizumab For Cytokine Release Syndrome. U.S. Food & Drug Administration. (2017). Available online at: https://www.fda.gov/drugs/resources-information-approved-drugs/fda-approves-tisagenlecleucel-b-cell-all-and-tocilizumab-cytokine-release-syndrome (accessed April 10, 2020).

[9] Brudno JN Kochenderfer JN. Toxicities of chimeric antigen receptor T cells: recognition and management. Blood. (2016) 127:3321–30. 10.1182/blood-2016-04-703751 27207799 PMC4929924

[10] Neelapu SS Tummala S Kebriaei P Wierda W Gutierrez C Locke FL. Chimeric antigen receptor T-cell therapy—assessment and management of toxicities. Nat Rev Clin Oncol. (2018) 15:47–62. 10.1038/nrclinonc.2017.148 28925994 PMC6733403

[11] Giavridis T van der Stegen SJC Eyquem J Hamieh M Piersigilli A Sadelain M. CAR T cell-induced cytokine release syndrome is mediated by macrophages and abated by IL-1 blockade. Nat Med. (2018) 24:731–8. 10.1038/s41591-018-0041-7 29808005 PMC6410714

[12] Greenbaum U Kebriaei P Srour SA Olson A Bashir Q Neelapu SS. Chimeric antigen receptor T-cell therapy toxicities. Br J Clin Pharmacol. (2020) bcp.14403. 10.1111/bcp.14403. [Epub ahead of print]. 32463929

[13] Richman SA Wang L-C Moon EK Khire UR Albelda SM Milone MC. Ligand-induced degradation of a CAR permits reversible remote control of CAR T cell activity *in vitro* and *in vivo*. Mol Ther. (2020) 28:1600–13. 10.1016/j.ymthe.2020.06.004 32559430 PMC7335755

[14] Brandt LJB Barnkob MB Michaels YS Heiselberg J Barington T. Emerging approaches for regulation and control of CAR T cells: a mini review. Front Immunol. (2020) 11:326. 10.3389/fimmu.2020.00326 32194561 PMC7062233

[15] Zhao J Lin Q Song Y Liu D. Universal CARs, universal T cells, and universal CAR T cells. J Hematol Oncol. (2018) 11:132. 10.1186/s13045-018-0677-2 30482221 PMC6257951

[16] Depil S Duchateau P Grupp SA Mufti G Poirot L. “Off-the-shelf” allogeneic CAR T cells: development and challenges. Nat Rev Drug Discov. (2020) 19:185–99. 10.1038/s41573-019-0051-2 31900462

[17] Ceppi F Rivers J Annesley C Pinto N Park JR Lindgren C. Lymphocyte apheresis for chimeric antigen receptor T-cell manufacturing in children and young adults with leukemia and neuroblastoma. Transfusion. (2018) 58:1414–20. 10.1111/trf.14569 29536556

[18] Walsh Z Ross S Fry TJ. Multi-specific CAR targeting to prevent antigen escape. Curr Hematol Malig Rep. (2019) 14:451–9. 10.1007/s11899-019-00537-5 31332617

[19] Provasi E Genovese P Lombardo A Magnani Z Liu P-Q Reik A. Editing T cell specificity towards leukemia by zinc finger nucleases and lentiviral gene transfer. Nat Med. (2012) 18:807–15. 10.1038/nm.2700 22466705 PMC5019824

[20] Torikai H Reik A Liu P-Q Zhou Y Zhang L Maiti S. A foundation for universal T-cell based immunotherapy: T cells engineered to express a CD19-specific chimeric-antigen-receptor and eliminate expression of endogenous TCR. Blood. (2012) 119:5697–705. 10.1182/blood-2012-01-405365 22535661 PMC3382929

[21] Torikai H Reik A Soldner F Warren EH Yuen C Zhou Y. Toward eliminating HLA class I expression to generate universal cells from allogeneic donors. Blood. (2013) 122:1341–9. 10.1182/blood-2013-03-478255 23741009 PMC3750336

[22] Poirot L Philip B Schiffer-Mannioui C Le Clerre D Chion-Sotinel I Derniame S. Multiplex genome-edited T-cell manufacturing platform for “Off-the-Shelf” adoptive T-cell immunotherapies. Cancer Res. (2015) 75:3853–64. 10.1158/0008-5472.CAN-14-3321 26183927

[23] Rasaiyaah J Georgiadis C Preece R Mock U Qasim W. TCRαβ/CD3 disruption enables CD3-specific antileukemic T cell immunotherapy. JCI insight. (2018) 3:e99442. 10.1172/jci.insight.99442 29997304 PMC6124532

[24] Sommer C Boldajipour B Kuo TC Bentley T Sutton J Chen A. Preclinical evaluation of allogeneic CAR T cells targeting BCMA for the treatment of multiple myeloma. Mol Ther. (2019) 27:1126–38. 10.1016/j.ymthe.2019.04.001 31005597 PMC6554542

[25] Ren J Liu X Fang C Jiang S June CH Zhao Y. Multiplex genome editing to generate universal CAR T cells resistant to PD1 inhibition. Clin Cancer Res. (2017) 23:2255–66. 10.1158/1078-0432.CCR-16-1300 27815355 PMC5413401

[26] Eyquem J Mansilla-Soto J Giavridis T van der Stegen SJC Hamieh M Cunanan KM. Targeting a CAR to the TRAC locus with CRISPR/Cas9 enhances tumour rejection. Nature. (2017) 543:113–7. 10.1038/nature21405 28225754 PMC5558614

[27] Georgiadis C Preece R Nickolay L Etuk A Petrova A Ladon D. Long terminal repeat CRISPR-CAR-coupled “Universal” T cells mediate potent anti-leukemic effects. Mol Ther. (2018) 26:1215–27. 10.1016/j.ymthe.2018.02.025 29605708 PMC5993944

[28] Jain N Graham C Konopleva M Yallop D Jozwik A Patten P. UCART19, an allogeneic anti- CD19 CAR T- cell product, in high risk adult patients with CD19+ relapsed/refractory B- cell acute lymphoblastic leukemia: preliminary results of phase I CALM study (Poster). EHA (2018). Available online at: https://learningcenter.ehaweb.org/eha/2018/stockholm/214674/reuben.benjamin.ucart19.an.allogeneic.anti-cd19.car.t-cell.product.in.high.html?~f=media=3*c_id=214674*listing=3*browseby=8 (accessed May 18, 2020).

[29] Valton J Guyot V Marechal A Filhol J-M Juillerat A Duclert A. A multidrug-resistant engineered CAR T cell for allogeneic combination immunotherapy. Mol Ther. (2015) 23:1507–18. 10.1038/mt.2015.104 26061646 PMC4817890

[30] Wang D Quan Y Yan Q Morales JE Wetsel RA. Targeted disruption of the β2-microglobulin gene minimizes the immunogenicity of human embryonic stem cells. Stem Cells Transl Med. (2015) 4:1234–45. 10.5966/sctm.2015-0049 26285657 PMC4572902

[31] Wilkie GM Taylor C Jones MM Burns DM Turner M Kilpatrick D. Establishment and characterization of a bank of cytotoxic T lymphocytes for immunotherapy of epstein-barr virus-associated diseases. J Immunother. (2004) 27:309–16. 10.1097/00002371-200407000-00007 15235392

[32] O'Reilly RJ Prockop S Hasan AN Koehne G Doubrovina E. Virus-specific T-cell banks for “off the shelf” adoptive therapy of refractory infections. Bone Marrow Transplant. (2016) 51:1163–72. 10.1038/bmt.2016.17 27042851 PMC5356365

[33] Withers B Clancy L Burgess J Simms R Brown R Micklethwaite K. Establishment and operation of a third-party virus-specific T cell bank within an allogeneic stem cell transplant program. Biol Blood Marrow Transplant. (2018) 24:2433–42. 10.1016/j.bbmt.2018.08.024 30172015

[34] Zhou L Liu X Wang X Sun Z Song X-T. CD123 redirected multiple virus-specific T cells for acute myeloid leukemia. Leuk Res. (2016) 41:76–84. 10.1016/j.leukres.2015.12.003 26740053

[35] Ostrom QT Gittleman H Liao P Rouse C Chen Y Dowling J. CBTRUS statistical report: primary brain and central nervous system tumors diagnosed in the United States in 2007-2011. Neuro Oncol. (2014) 16:iv1–63. 10.1093/neuonc/nou223 25304271 PMC4193675

[36] Weller M Wick W Aldape K Brada M Berger M Pfister SM. Glioma. Nat Rev Dis Prim. (2015) 1:15017. 10.1038/nrdp.2015.17 27188790

[37] Stupp R Hegi ME Mason WP van den Bent MJ Taphoorn MJB Janzer RC. Effects of radiotherapy with concomitant and adjuvant temozolomide versus radiotherapy alone on survival in glioblastoma in a randomised phase III study: 5-year analysis of the EORTC-NCIC trial. Lancet Oncol. (2009) 10:459–66. 10.1016/S1470-2045(09)70025-7 19269895

[38] Marenco-Hillembrand L Wijesekera O Suarez-Meade P Mampre D Jackson C Peterson J. Trends in glioblastoma: outcomes over time and type of intervention: a systematic evidence based analysis. J Neurooncol. (2020) 147:297–307. 10.1007/s11060-020-03451-6 32157552

[39] Fecci PE Sampson JH. The current state of immunotherapy for gliomas: an eye toward the future. J Neurosurg. (2019) 131:657–66. 10.3171/2019.5.JNS181762 31473668

[40] Dutoit V Migliorini D Dietrich P-Y. Current strategies for vaccination in glioblastoma. Curr Opin Oncol. (2019) 31:514–21. 10.1097/CCO.0000000000000575 31403483

[41] Medikonda R Dunn G Rahman M Fecci P Lim M. A review of glioblastoma immunotherapy. J Neurooncol. (2021) 151:41–53. 10.1007/s11060-020-03448-1 32253714

[42] Brahm CG van Linde ME Enting RH Schuur M Otten RHJ Heymans MW. The current status of immune checkpoint inhibitors in neuro-oncology: a systematic review. Cancers (Basel). (2020) 12:586. 10.3390/cancers12030586 32143288 PMC7139638

[43] Chheda ZS Kohanbash G Okada K Jahan N Sidney J Pecoraro M. Novel and shared neoantigen derived from histone 3 variant H3.3K27M mutation for glioma T cell therapy. J Exp Med. (2018) 215:141–57. 10.1084/jem.20171046 29203539 PMC5748856

[44] Keskin DB Anandappa AJ Sun J Tirosh I Mathewson ND Li S. Neoantigen vaccine generates intratumoral T cell responses in phase Ib glioblastoma trial. Nature. (2019) 565:234–9. 10.1038/s41586-018-0792-9 30568305 PMC6546179

[45] Migliorini D Dutoit V Allard M Grandjean Hallez N Marinari E Widmer V. Phase I/II trial testing safety and immunogenicity of the multipeptide IMA950/poly-ICLC vaccine in newly diagnosed adult malignant astrocytoma patients. Neuro Oncol. (2019) 21:923–33. 10.1093/neuonc/noz040 30753611 PMC6620642

[46] Hilf N Kuttruff-Coqui S Frenzel K Bukur V Stevanović S Gouttefangeas C. Actively personalized vaccination trial for newly diagnosed glioblastoma. Nature. (2019) 565:240–5. 10.1038/s41586-018-0810-y 30568303

[47] Cloughesy TF Mochizuki AY Orpilla JR Hugo W Lee AH Davidson TB. Neoadjuvant anti-PD-1 immunotherapy promotes a survival benefit with intratumoral and systemic immune responses in recurrent glioblastoma. Nat Med. (2019) 25:477–86. 10.1038/s41591-018-0337-7 30742122 PMC6408961

[48] Schalper KA Rodriguez-Ruiz ME Diez-Valle R López-Janeiro A Porciuncula A Idoate MA. Neoadjuvant nivolumab modifies the tumor immune microenvironment in resectable glioblastoma. Nat Med. (2019) 25:470–6. 10.1038/s41591-018-0339-5 30742120

[49] Zhao J Chen AX Gartrell RD Silverman AM Aparicio L Chu T. Immune and genomic correlates of response to anti-PD-1 immunotherapy in glioblastoma. Nat Med. (2019) 25:462–9. 10.1038/s41591-019-0349-y 30742119 PMC6810613

[50] Migliorini D Dietrich P-Y Stupp R Linette GP Posey AD June CH. CAR T-cell therapies in glioblastoma: a first look. Clin Cancer Res. (2018) 24:535–40. 10.1158/1078-0432.CCR-17-2871 29158268

[51] Prinzing BL Gottschalk SM Krenciute G. CAR T-cell therapy for glioblastoma: ready for the next round of clinical testing? Expert Rev Anticancer Ther. (2018) 18:451–61. 10.1080/14737140.2018.1451749 29533108 PMC6191291

[52] Brown CE Alizadeh D Starr R Weng L Wagner JR Naranjo A. Regression of glioblastoma after chimeric antigen receptor T-cell therapy. N Engl J Med. (2016) 375:2561–9. 10.1056/NEJMoa1610497 28029927 PMC5390684

[53] Ahmed N Brawley V Hegde M Bielamowicz K Kalra M Landi D. HER2-specific chimeric antigen receptor-modified virus-specific T cells for progressive glioblastoma: a phase 1 dose-escalation trial. JAMA Oncol. (2017) 3:1094–101. 10.1001/jamaoncol.2017.0184 28426845 PMC5747970

[54] O'Rourke DM Nasrallah MP Desai A Melenhorst JJ Mansfield K Morrissette JJD. A single dose of peripherally infused EGFRvIII-directed CAR T cells mediates antigen loss and induces adaptive resistance in patients with recurrent glioblastoma. Sci Transl Med. (2017) 9:eaaa0984. 10.1126/scitranslmed.aaa0984 28724573 PMC5762203

[55] McCreedy BJ Senyukov VV Nguyen KT. Off the shelf T cell therapies for hematologic malignancies. Best Pract Res Clin Haematol. (2018) 31:166–75. 10.1016/j.beha.2018.03.001 29909917

[56] Kaminski BA Kadereit S Miller RE Leahy P Stein KR Topa DA. Reduced expression of NFAT-associated genes in UCB versus adult CD4+ T lymphocytes during primary stimulation. Blood. (2003) 102:4608–17. 10.1182/blood-2003-05-1732 12946996

[57] Nitsche A Zhang M Clauss T Siegert W Brune K Pahl A. Cytokine profiles of cord and adult blood leukocytes: differences in expression are due to differences in expression and activation of transcription factors. BMC Immunol. (2007) 8:18. 10.1186/1471-2172-8-18 17764543 PMC2018703

[58] Gutman JA Ross K Smith C Myint H Lee C-K Salit R. Chronic graft versus host disease burden and late transplant complications are lower following adult double cord blood versus matched unrelated donor peripheral blood transplantation. Bone Marrow Transplant. (2016) 51:1588–93. 10.1038/bmt.2016.186 27400068

[59] Li D Li X Liao L Li N. Unrelated cord blood transplantation versus haploidentical transplantation in adult and pediatric patients with hematological malignancies-A meta-analysis and systematic review. Am J Blood Res. (2020) 10:1–10. 32206440 PMC7076284

[60] Sharma P Purev E Haverkos B Pollyea DA Cherry E Kamdar M. Adult cord blood transplant results in comparable overall survival and improved GRFS vs. matched related transplant. Blood Adv. (2020) 4:2227–35. 10.1182/bloodadvances.2020001554 32442301 PMC7252552

[61] Ma Q Garber HR Lu S He H Tallis E Ding X. A novel TCR-like CAR with specificity for PR1/HLA-A2 effectively targets myeloid leukemia *in vitro* when expressed in human adult peripheral blood and cord blood T cells. Cytotherapy. (2016) 18:985–94. 10.1016/j.jcyt.2016.05.001 27265873 PMC4935572

[62] Papapetrou EP. Induced pluripotent stem cells, past and future. Science. (2016) 353:991–2. 10.1126/science.aai7626 27701103 PMC5234330

[63] Themeli M Kloss CC Ciriello G Fedorov VD Perna F Gonen M. Generation of tumor-targeted human T lymphocytes from induced pluripotent stem cells for cancer therapy. Nat Biotechnol. (2013) 31:928–33. 10.1038/nbt.2678 23934177 PMC5722218

[64] Styczyński J Tridello G Koster L Iacobelli S van Biezen A van der Werf S. Death after hematopoietic stem cell transplantation: changes over calendar year time, infections and associated factors. Bone Marrow Transplant. (2020) 55:126–36. 10.1038/s41409-019-0624-z 31455899 PMC6957465

[65] Jagasia MH Greinix HT Arora M Williams KM Wolff D Cowen EW. National institutes of health consensus development project on criteria for clinical trials in chronic graft-versus-host disease: I. The 2014 diagnosis and staging working group report. Biol Blood Marrow Transplant. (2015) 21:389–401.e1. 10.1016/j.bbmt.2014.12.001 25529383 PMC4329079

[66] Abdelhakim H Abdel-Azim H Saad A. Role of αβ T cell depletion in prevention of graft versus host disease. Biomedicines. (2017) 5:35. 10.3390/biomedicines5030035 28672883 PMC5618293

[67] Melenhorst JJ Leen AM Bollard CM Quigley MF Price DA Rooney CM. Allogeneic virus-specific T cells with HLA alloreactivity do not produce GVHD in human subjects. Blood. (2010) 116:4700–2. 10.1182/blood-2010-06-289991 20709906 PMC2996125

[68] Withers B Blyth E Clancy LE Yong A Fraser C Burgess J. Long-term control of recurrent or refractory viral infections after allogeneic HSCT with third-party virus-specific T cells. Blood Adv. (2017) 1:2193–205. 10.1182/bloodadvances.2017010223 29296867 PMC5737128

[69] Tzannou I Papadopoulou A Naik S Leung K Martinez CA Ramos CA. Off-the-shelf virus-specific T cells to treat BK virus, human herpesvirus 6, cytomegalovirus, epstein-barr virus, and adenovirus infections after allogeneic hematopoietic stem-cell transplantation. J Clin Oncol. (2017) 35:3547–57. 10.1200/JCO.2017.73.0655 28783452 PMC5662844

[70] D'Orsogna LJA Roelen DL Doxiadis IIN Claas FHJ. Alloreactivity from human viral specific memory T-cells. Transpl Immunol. (2010) 23:149–55. 10.1016/j.trim.2010.06.008 20600900

[71] Cruz CRY Micklethwaite KP Savoldo B Ramos CA Lam S Ku S. Infusion of donor-derived CD19-redirected virus-specific T cells for B-cell malignancies relapsed after allogeneic stem cell transplant: a phase 1 study. Blood. (2013) 122:2965–73. 10.1182/blood-2013-06-506741 24030379 PMC3811171

[72] Münz C. Redirecting T cells against epstein–barr virus infection and associated oncogenesis. Cells. (2020) 9:1400. 10.3390/cells9061400 32512847 PMC7349826

[73] Okamoto S Mineno J Ikeda H Fujiwara H Yasukawa M Shiku H. Improved expression and reactivity of transduced tumor-specific TCRs in human lymphocytes by specific silencing of endogenous TCR. Cancer Res. (2009) 69:9003–11. 10.1158/0008-5472.CAN-09-1450 19903853

[74] Berdien B Mock U Atanackovic D Fehse B. TALEN-mediated editing of endogenous T-cell receptors facilitates efficient reprogramming of T lymphocytes by lentiviral gene transfer. Gene Ther. (2014) 21:539–48. 10.1038/gt.2014.26 24670996

[75] Osborn MJ Webber BR Knipping F Lonetree C Tennis N DeFeo AP. Evaluation of TCR gene editing achieved by TALENs, CRISPR/Cas9, and megaTAL nucleases. Mol Ther. (2016) 24:570–81. 10.1038/mt.2015.197 26502778 PMC4786913

[76] MacLeod DT Antony J Martin AJ Moser RJ Hekele A Wetzel KJ. Integration of a CD19 CAR into the TCR alpha chain locus streamlines production of allogeneic gene-edited CAR T cells. Mol Ther. (2017) 25:949–61. 10.1016/j.ymthe.2017.02.005 28237835 PMC5383629

[77] Wiebking V Lee CM Mostrel N Lahiri P Bak R Bao G. Genome editing of donor-derived T-cells to generate allogenic chimeric antigen receptor-modified T cells: optimizing αβ T cell-depleted haploidentical hematopoietic stem cell transplantation. Haematologica. (2020). 10.3324/haematol.2019.233882. [Epub ahead of print]. PMC7928014 32241852

[78] Ren J Zhang X Liu X Fang C Jiang S June CH. A versatile system for rapid multiplex genome-edited CAR T cell generation. Oncotarget. (2017) 8:17002–11. 10.18632/oncotarget.15218 28199983 PMC5370017

[79] Fraietta JA Nobles CL Sammons MA Lundh S Carty SA Reich TJ. Disruption of TET2 promotes the therapeutic efficacy of CD19-targeted T cells. Nature. (2018) 558:307–12. 10.1038/s41586-018-0178-z 29849141 PMC6320248

[80] Bothmer A Gareau KW Abdulkerim HS Buquicchio F Cohen L Viswanathan R. Detection and modulation of DNA translocations during multi-gene genome editing in T cells. Cris J. (2020) 3:177–87. 10.1089/crispr.2019.0074 32584143

[81] Hale M Lee B Honaker Y Leung W-H Grier AE Jacobs HM. Homology-directed recombination for enhanced engineering of chimeric antigen receptor T cells. Mol Ther Methods Clin Dev. (2017) 4:192–203. 10.1016/j.omtm.2016.12.008 28345004 PMC5363294

[82] Roth TL Puig-Saus C Yu R Shifrut E Carnevale J Li PJ. Reprogramming human T cell function and specificity with non-viral genome targeting. Nature. (2018) 559:405–9. 10.1038/s41586-018-0326-5 29995861 PMC6239417

[83] Schober K Müller TR Gökmen F Grassmann S Effenberger M Poltorak M. Orthotopic replacement of T-cell receptor α- and β-chains with preservation of near-physiological T-cell function. Nat Biomed Eng. (2019) 3:974–84. 10.1038/s41551-019-0409-0 31182835

[84] Rotolo R Leuci V Donini C Cykowska A Gammaitoni L Medico G. CAR-Based strategies beyond T lymphocytes: integrative opportunities for cancer adoptive immunotherapy. Int J Mol Sci. (2019) 20:2839. 10.3390/ijms20112839 31212634 PMC6600566

[85] Petty AJ Heyman B Yang Y. Chimeric Antigen receptor cell therapy: overcoming obstacles to battle cancer. Cancers (Basel). (2020) 12:842. 10.3390/cancers12040842 32244520 PMC7226583

[86] Mehta RS Rezvani K. Chimeric antigen receptor expressing natural killer cells for the immunotherapy of cancer. Front Immunol. (2018) 9:283. 10.3389/fimmu.2018.00283 29497427 PMC5818392

[87] Saetersmoen ML Hammer Q Valamehr B Kaufman DS Malmberg K-J. Off-the-shelf cell therapy with induced pluripotent stem cell-derived natural killer cells. Semin Immunopathol. (2019) 41:59–68. 10.1007/s00281-018-0721-x 30361801

[88] Rotolo A Caputo VS Holubova M Baxan N Dubois O Chaudhry MS. Enhanced anti-lymphoma activity of CAR19-iNKT cells underpinned by dual CD19 and CD1d targeting. Cancer Cell. (2018) 34:596–610.e11. 10.1016/j.ccell.2018.08.017 30300581 PMC6179961

[89] Xu X Huang W Heczey A Liu D Guo L Wood M. NKT cells coexpressing a GD2-specific chimeric antigen receptor and IL15 show enhanced *in vivo* persistence and antitumor activity against neuroblastoma. Clin Cancer Res. (2019) 25:7126–38. 10.1158/1078-0432.CCR-19-0421 31484667 PMC6891170

[90] Capsomidis A Benthall G Van Acker HH Fisher J Kramer AM Abeln Z. Chimeric antigen receptor-engineered human gamma delta T cells: enhanced cytotoxicity with retention of cross presentation. Mol Ther. (2018) 26:354–65. 10.1016/j.ymthe.2017.12.001 29310916 PMC5835118

[91] Zeng J Tang SY Wang S. Derivation of mimetic γδ T cells endowed with cancer recognition receptors from reprogrammed γδ T cell. PLoS ONE. (2019) 14:e0216815. 10.1371/journal.pone.0216815 31071196 PMC6508724

[92] Yazdanifar M Barbarito G Bertaina A Airoldi I. γδ T cells: the ideal tool for cancer immunotherapy. Cells. (2020) 9:1305. 10.3390/cells9051305 32456316 PMC7290982

[93] Anwer F Shaukat A-A Zahid U Husnain M McBride A Persky D. Donor origin CAR T cells: graft versus malignancy effect without GVHD, a systematic review. Immunotherapy. (2017) 9:123–30. 10.2217/imt-2016-0127 28128714 PMC5827793

[94] Maude SL Frey N Shaw PA Aplenc R Barrett DM Bunin NJ. Chimeric antigen receptor T cells for sustained remissions in leukemia. N Engl J Med. (2014) 371:1507–17. 10.1056/NEJMoa1407222 25317870 PMC4267531

[95] Fraietta JA Lacey SF Orlando EJ Pruteanu-Malinici I Gohil M Lundh S. Determinants of response and resistance to CD19 chimeric antigen receptor (CAR) T cell therapy of chronic lymphocytic leukemia. Nat Med. (2018) 24:563–71. 10.1038/s41591-018-0010-1 29713085 PMC6117613

[96] Louis CU Savoldo B Dotti G Pule M Yvon E Myers GD. Antitumor activity and long-term fate of chimeric antigen receptor-positive T cells in patients with neuroblastoma. Blood. (2011) 118:6050–6. 10.1182/blood-2011-05-354449 21984804 PMC3234664

[97] Benjamin R Graham C Yallop D Jozwik A Ciocarlie O Jain N. Preliminary data on safety, cellular kinetics and anti-leukemic activity of UCART19, an allogeneic anti-CD19 CAR T-cell product, in a pool of adult and pediatric patients with high-risk CD19+ relapsed/refractory B-cell acute lymphoblastic leukemia. Blood. (2018) 132:896. 10.1182/blood-2018-99-111356

[98] Zheng D Wang X Xu R-H. Concise review: one stone for multiple birds: generating universally compatible human embryonic stem cells. Stem Cells. (2016) 34:2269–75. 10.1002/stem.2407 27251112

[99] Zhao W Lei A Tian L Wang X Correia C Weiskittel T. Strategies for genetically engineering hypoimmunogenic universal pluripotent stem cells. iScience. (2020) 23:101162. 10.1016/j.isci.2020.101162 32502965 PMC7270609

[100] Revenfeld ALS Steffensen R Pugholm LH Jørgensen MM Stensballe A Varming K. Presence of HLA-DR molecules and HLA-DRB1 mRNA in circulating CD4(+) T cells. Scand J Immunol. (2016) 84:211–21. 10.1111/sji.12462 27417521

[101] Holling TM van der Stoep N Quinten E van den Elsen PJ. Activated human T cells accomplish MHC class II expression through T cell-specific occupation of class II transactivator promoter III. J Immunol. (2002) 168:763–70. 10.4049/jimmunol.168.2.763 11777970

[102] Krawczyk M Peyraud N Rybtsova N Masternak K Bucher P Barras E. Long distance control of MHC class II expression by multiple distal enhancers regulated by regulatory factor X complex and CIITA. J Immunol. (2004) 173:6200–10. 10.4049/jimmunol.173.10.6200 15528357

[103] Kagoya Y Guo T Yeung B Saso K Anczurowski M Wang C-H. Genetic ablation of HLA class I, class II, and the T-cell receptor enables allogeneic T cells to be used for adoptive T-cell therapy. Cancer Immunol Res. (2020) 8:926–36. 10.1158/2326-6066.CIR-18-0508 32321775

[104] de Rham C Calderin Sollet Z Burkhard P Villard J. Natural killer cell alloreactivity against human induced pluripotent stem cells and their neuronal derivatives into dopaminergic neurons. Stem Cells Dev. (2020) 29:853–62. 10.1089/scd.2019.0201 32245345

[105] Farnault L Sanchez C Baier C Le Treut T Costello RT. Hematological malignancies escape from NK cell innate immune surveillance: mechanisms and therapeutic implications. Clin Dev Immunol. (2012) 2012:1–8. 10.1155/2012/421702 22899948 PMC3415262

[106] Baier C Fino A Sanchez C Farnault L Rihet P Kahn-Perlès B. Natural killer cells modulation in hematological malignancies. Front Immunol. (2013) 4:459. 10.3389/fimmu.2013.00459 24391641 PMC3867693

[107] Kochan G Escors D Breckpot K Guerrero-Setas D. Role of non-classical MHC class I molecules in cancer immunosuppression. Oncoimmunology. (2013) 2:e26491. 10.4161/onci.26491 24482746 PMC3894240

[108] Gornalusse GG Hirata RK Funk SE Riolobos L Lopes VS Manske G. HLA-E-expressing pluripotent stem cells escape allogeneic responses and lysis by NK cells. Nat Biotechnol. (2017) 35:765–72. 10.1038/nbt.3860 28504668 PMC5548598

[109] Carosella ED Rouas-Freiss N Roux DT-L Moreau P LeMaoult J. HLA-G in immune checkpoint molecule. Adv Immunol. (2015) 127:33–144. 10.1016/bs.ai.2015.04.001 26073983

[110] Jandus C Boligan KF Chijioke O Liu H Dahlhaus M Démoulins T. Interactions between Siglec-7/9 receptors and ligands influence NK cell-dependent tumor immunosurveillance. J Clin Invest. (2014) 124:1810–20. 10.1172/JCI65899 24569453 PMC3973073

[111] Zheng Y Ma X Su D Zhang Y Yu L Jiang F. The roles of Siglec7 and Siglec9 on natural killer cells in virus infection and tumour progression. J Immunol Res. (2020) 2020:1–9. 10.1155/2020/6243819 32322597 PMC7165337

[112] Mo F Watanabe N McKenna MK Hicks MJ Srinivasan M Gomes-Silva D. Engineered off-the-shelf therapeutic T cells resist host immune rejection. Nat Biotechnol. (2020) 39:56–63. 10.1038/s41587-020-0601-5 32661440 PMC7854790

[113] Majzner RG Mackall CL. Tumor antigen escape from CAR T-cell therapy. Cancer Discov. (2018) 8:1219–26. 10.1158/2159-8290.CD-18-0442 30135176

[114] Ruella M Maus MV. Catch me if you can: leukemia escape after CD19-directed T cell immunotherapies. Comput Struct Biotechnol J. (2016) 14:357–62. 10.1016/j.csbj.2016.09.003 27761200 PMC5061074

[115] Anurathapan U Chan RC Hindi HF Mucharla R Bajgain P Hayes BC. Kinetics of tumor destruction by chimeric antigen receptor-modified T cells. Mol Ther. (2014) 22:623–33. 10.1038/mt.2013.262 24213558 PMC3945803

[116] Minutolo NG Hollander EE Powell DJ. The emergence of universal immune receptor T cell therapy for cancer. Front Oncol. (2019) 9:176. 10.3389/fonc.2019.00176 30984613 PMC6448045

[117] Hughes-Parry HE Cross RS Jenkins MR. The evolving protein engineering in the design of chimeric antigen receptor T cells. Int J Mol Sci. (2019) 21:204. 10.3390/ijms21010204 31892219 PMC6981602

[118] Cho JH Collins JJ Wong WW. Universal chimeric antigen receptors for multiplexed and logical control of T cell responses. Cell. (2018) 173:1426–38.e11. 10.1016/j.cell.2018.03.038 29706540 PMC5984158

[119] Cartellieri M Feldmann A Koristka S Arndt C Loff S Ehninger A. Switching CAR T cells on and off: a novel modular platform for retargeting of T cells to AML blasts. Blood Cancer J. (2016) 6:e458. 10.1038/bcj.2016.61 27518241 PMC5022178

[120] Bachmann M. The UniCAR system: a modular CAR T cell approach to improve the safety of CAR T cells. Immunol Lett. (2019) 211:13–22. 10.1016/j.imlet.2019.05.003 31091431

[121] Feldmann A Arndt C Koristka S Berndt N Bergmann R Bachmann MP. Conventional CARs versus modular CARs. Cancer Immunol Immunother. (2019) 68:1713–9. 10.1007/s00262-019-02399-5 31542798 PMC6805801

[122] Darowski D Kobold S Jost C Klein C. Combining the best of two worlds: highly flexible chimeric antigen receptor adaptor molecules (CAR-adaptors) for the recruitment of chimeric antigen receptor T cells. MAbs. (2019) 11:621–31. 10.1080/19420862.2019.1596511 30892136 PMC6601549

[123] Minutolo NG Sharma P Poussin M Shaw LC Brown DP Hollander EE. Quantitative control of gene-engineered T-cell activity through the covalent attachment of targeting ligands to a universal immune receptor. J Am Chem Soc. (2020) 142:6554–68. 10.1021/jacs.9b11622 32191035 PMC7306176

[124] Liu D Zhao J Song Y. Engineering switchable and programmable universal CARs for CAR T therapy. J Hematol Oncol. (2019) 12:69. 10.1186/s13045-019-0763-0 31272471 PMC6610960

[125] Veluchamy JP Kok N van der Vliet HJ Verheul HMW de Gruijl TD Spanholtz J. The rise of allogeneic natural killer cells as a platform for cancer immunotherapy: recent innovations and future developments. Front Immunol. (2017) 8:631. 10.3389/fimmu.2017.00631 28620386 PMC5450018

[126] Pfefferle A Huntington ND. You have got a fast CAR: chimeric antigen receptor NK cells in cancer therapy. Cancers (Basel). (2020) 12:706. 10.3390/cancers12030706 PMC7140022 32192067

[127] Shin MH Kim J Lim SA Kim J Kim S-J Lee K-M. NK cell-based immunotherapies in cancer. Immune Netw. (2020) 20:e14. 10.4110/in.2020.20.e14 32395366 PMC7192832

[128] Maude SL Barrett D Teachey DT Grupp SA. Managing cytokine release syndrome associated with novel T cell-engaging therapies. Cancer J. (2014) 20:119–22. 10.1097/PPO.0000000000000035 24667956 PMC4119809

[129] Davila ML Riviere I Wang X Bartido S Park J Curran K. Efficacy and toxicity management of 19-28z CAR T cell therapy in B cell acute lymphoblastic leukemia. Sci Transl Med. (2014) 6:224ra25. 10.1126/scitranslmed.3008226 24553386 PMC4684949

[130] Singh N Hofmann TJ Gershenson Z Levine BL Grupp SA Teachey DT. Monocyte lineage-derived IL-6 does not affect chimeric antigen receptor T-cell function. Cytotherapy. (2017) 19:867–80. 10.1016/j.jcyt.2017.04.001 28506444 PMC6676485

[131] Liu M Meng Y Zhang L Han Z Feng X. High-efficient generation of natural killer cells from peripheral blood with preferable cell vitality and enhanced cytotoxicity by combination of IL-2, IL-15 and IL-18. Biochem Biophys Res Commun. (2021) 534:149–56. 10.1016/j.bbrc.2020.12.012 33309274

[132] Daher M Basar R Gokdemir E Baran N Uprety N Nunez Cortes AK. Targeting a cytokine checkpoint enhances the fitness of armored cord blood CAR-NK cells. Blood. (2021) 137:624–36. 10.1182/blood.2020007748 32902645 PMC7869185

[133] Suck G Odendahl M Nowakowska P Seidl C Wels WS Klingemann HG. NK-92: an “off-the-shelf therapeutic” for adoptive natural killer cell-based cancer immunotherapy. Cancer Immunol Immunother. (2016) 65:485–92. 10.1007/s00262-015-1761-x 26559813 PMC11029582

[134] Liu E Marin D Banerjee P Macapinlac HA Thompson P Basar R. Use of CAR-transduced natural killer cells in CD19-positive lymphoid tumors. N Engl J Med. (2020) 382:545–53. 10.1056/NEJMoa1910607 32023374 PMC7101242

[135] Pascal V Schleinitz N Brunet C Ravet S Bonnet E Lafarge X. Comparative analysis of NK cell subset distribution in normal and lymphoproliferative disease of granular lymphocyte conditions. Eur J Immunol. (2004) 34:2930–40. 10.1002/eji.200425146 15368309

[136] Angelo LS Banerjee PP Monaco-Shawver L Rosen JB Makedonas G Forbes LR. Practical NK cell phenotyping and variability in healthy adults. Immunol Res. (2015) 62:341–56. 10.1007/s12026-015-8664-y 26013798 PMC4470870

[137] Zhang C Oberoi P Oelsner S Waldmann A Lindner A Tonn T. Chimeric antigen receptor-engineered NK-92 cells: an off-the-shelf cellular therapeutic for targeted elimination of cancer cells and induction of protective antitumor immunity. Front Immunol. (2017) 8:533. 10.3389/fimmu.2017.00533 28572802 PMC5435757

[138] Huang R-S Shih H-A Lai M-C Chang Y-J Lin S. Enhanced NK-92 cytotoxicity by CRISPR genome engineering using Cas9 ribonucleoproteins. Front Immunol. (2020) 11:1008. 10.3389/fimmu.2020.01008 32528479 PMC7256201

[139] Mitwasi N Feldmann A Arndt C Koristka S Berndt N Jureczek J. “UniCAR”-modified off-the-shelf NK-92 cells for targeting of GD2-expressing tumour cells. Sci Rep. (2020) 10:2141. 10.1038/s41598-020-59082-4 32034289 PMC7005792

[140] Zhang C Burger MC Jennewein L Genßler S Schönfeld K Zeiner P. ErbB2/HER2-specific NK cells for targeted therapy of glioblastoma. JNCI J Natl Cancer Inst. (2016) 108. 10.1093/jnci/djv375 26640245

[141] Nowakowska P Romanski A Miller N Odendahl M Bonig H Zhang C. Clinical grade manufacturing of genetically modified, CAR-expressing NK-92 cells for the treatment of ErbB2-positive malignancies. Cancer Immunol Immunother. (2018) 67:25–38. 10.1007/s00262-017-2055-2 28879551 PMC11028154

[142] Burger MC Zhang C Harter PN Romanski A Strassheimer F Senft C. CAR-engineered NK cells for the treatment of glioblastoma: turning innate effectors into precision tools for cancer immunotherapy. Front Immunol. (2019) 10:2683. 10.3389/fimmu.2019.02683 31798595 PMC6868035

[143] Montagner IM Penna A Fracasso G Carpanese D Dalla Pietà A Barbieri V. Anti-PSMA CAR-engineered NK-92 cells: an off-the-shelf cell therapy for prostate cancer. Cells. (2020) 9:1382. 10.3390/cells9061382 32498368 PMC7349573

[144] Heczey A Liu D Tian G Courtney AN Wei J Marinova E. Invariant NKT cells with chimeric antigen receptor provide a novel platform for safe and effective cancer immunotherapy. Blood. (2014) 124:2824–33. 10.1182/blood-2013-11-541235 25049283 PMC4215313

[145] Godfrey DI Kronenberg M. Going both ways: immune regulation *via* CD1d-dependent NKT cells. J Clin Invest. (2004) 114:1379–88. 10.1172/JCI23594 15545985 PMC525753

[146] Carreño LJ Saavedra-Ávila NA Porcelli SA. Synthetic glycolipid activators of natural killer T cells as immunotherapeutic agents. Clin Transl Immunol. (2016) 5:e69. 10.1038/cti.2016.14 27195112 PMC4855264

[147] Chaidos A Patterson S Szydlo R Chaudhry MS Dazzi F Kanfer E. Graft invariant natural killer T-cell dose predicts risk of acute graft-versus-host disease in allogeneic hematopoietic stem cell transplantation. Blood. (2012) 119:5030–6. 10.1182/blood-2011-11-389304 22371885 PMC6143158

[148] Coman T Rossignol J D'Aveni M Fabiani B Dussiot M Rignault R. Human CD4- invariant NKT lymphocytes regulate graft versus host disease. Oncoimmunology. (2018) 7:e1470735. 10.1080/2162402X.2018.1470735 30377560 PMC6204997

[149] Tian G Courtney AN Jena B Heczey A Liu D Marinova E. CD62L+ NKT cells have prolonged persistence and antitumor activity *in vivo*. J Clin Invest. (2016) 126:2341–55. 10.1172/JCI83476 27183388 PMC4887157

[150] Patel S Burga RA Powell AB Chorvinsky EA Hoq N McCormack SE. Beyond CAR T cells: other cell-based immunotherapeutic strategies against cancer. Front Oncol. (2019) 9:196. 10.3389/fonc.2019.00196 31024832 PMC6467966

[151] Engelhardt B Ransohoff RM. Capture, crawl, cross: the T cell code to breach the blood-brain barriers. Trends Immunol. (2012) 33:579–89. 10.1016/j.it.2012.07.004 22926201

[152] Salinas RD Durgin JS O'Rourke DM. Potential of glioblastoma-targeted chimeric antigen receptor (CAR) T-cell therapy. CNS Drugs. (2020) 34:127–45. 10.1007/s40263-019-00687-3 31916100

[153] Sackstein R Schatton T Barthel SR. T-lymphocyte homing: an underappreciated yet critical hurdle for successful cancer immunotherapy. Lab Invest. (2017) 97:669–97. 10.1038/labinvest.2017.25 28346400 PMC5446300

[154] Brown CE Aguilar B Starr R Yang X Chang W-C Weng L. Optimization of IL13Rα2-targeted chimeric antigen receptor T cells for improved anti-tumor efficacy against glioblastoma. Mol Ther. (2018) 26:31–44. 10.1016/j.ymthe.2017.10.002 29103912 PMC5763077

[155] Theruvath J Sotillo E Mount CW Graef CM Delaidelli A Heitzeneder S. Locoregionally administered B7-H3-targeted CAR T cells for treatment of atypical teratoid/rhabdoid tumors. Nat Med. (2020) 26:712–9. 10.1038/s41591-020-0821-8 32341579 PMC7992505

[156] Donovan LK Delaidelli A Joseph SK Bielamowicz K Fousek K Holgado BL. Locoregional delivery of CAR T cells to the cerebrospinal fluid for treatment of metastatic medulloblastoma and ependymoma. Nat Med. (2020) 26:720–31. 10.1038/s41591-020-0827-2 32341580 PMC8815773

[157] Tian Y Li Y Shao Y Zhang Y. Gene modification strategies for next-generation CAR T cells against solid cancers. J Hematol Oncol. (2020) 13:54. 10.1186/s13045-020-00890-6 32423475 PMC7236186

[158] Jin L Tao H Karachi A Long Y Hou AY Na M. CXCR1- or CXCR2-modified CAR T cells co-opt IL-8 for maximal antitumor efficacy in solid tumors. Nat Commun. (2019) 10:4016. 10.1038/s41467-019-11869-4 31488817 PMC6728370

[159] Alfaro C Teijeira A Oñate C Pérez G Sanmamed MF Andueza MP. Tumor-produced interleukin-8 attracts human myeloid-derived suppressor cells and elicits extrusion of neutrophil extracellular traps (NETs). Clin Cancer Res. (2016) 22:3924–36. 10.1158/1078-0432.CCR-15-2463 26957562

[160] Fernando RI Castillo MD Litzinger M Hamilton DH Palena C. IL-8 signaling plays a critical role in the epithelial-mesenchymal transition of human carcinoma cells. Cancer Res. (2011) 71:5296–306. 10.1158/0008-5472.CAN-11-0156 21653678 PMC3148346

[161] Oelkrug C Ramage JM. Enhancement of T cell recruitment and infiltration into tumours. Clin Exp Immunol. (2014) 178:1–8. 10.1111/cei.12382 24828133 PMC4360188

[162] Brown CE Vishwanath RP Aguilar B Starr R Najbauer J Aboody KS. Tumor-derived chemokine MCP-1/CCL2 is sufficient for mediating tumor tropism of adoptively transferred T cells. J Immunol. (2007) 179:3332–41. 10.4049/jimmunol.179.5.3332 17709550

[163] Liu C Luo D Reynolds BA Meher G Katritzky AR Lu B. Chemokine receptor CXCR3 promotes growth of glioma. Carcinogenesis. (2011) 32:129–37. 10.1093/carcin/bgq224 21051441 PMC3026840

[164] Mikucki ME Fisher DT Matsuzaki J Skitzki JJ Gaulin NB Muhitch JB. Non-redundant requirement for CXCR3 signalling during tumoricidal T-cell trafficking across tumour vascular checkpoints. Nat Commun. (2015) 6:7458. 10.1038/ncomms8458 26109379 PMC4605273

[165] Thust SC van den Bent MJ Smits M. Pseudoprogression of brain tumors. J Magn Reson Imaging. (2018) 48:571–89. 10.1002/jmri.26171 PMC6175399 29734497

[166] Sanghera P Perry J Sahgal A Symons S Aviv R Morrison M. Pseudoprogression following chemoradiotherapy for glioblastoma multiforme. Can J Neurol Sci. (2010) 37:36–42. 10.1017/s0317167100009628 20169771

[167] Chapelin F Capitini CM Ahrens ET. Fluorine-19 MRI for detection and quantification of immune cell therapy for cancer. J Immunother cancer. (2018) 6:105. 10.1186/s40425-018-0416-9 30305175 PMC6180584

[168] Kircher MF Gambhir SS Grimm J. Noninvasive cell-tracking methods. Nat Rev Clin Oncol. (2011) 8:677–88. 10.1038/nrclinonc.2011.141 21946842

[169] McCracken MN Tavaré R Witte ON Wu AM. Advances in PET detection of the antitumor T cell response. Adv Immunol. (2016) 131:187–231. 10.1016/bs.ai.2016.02.004 27235684 PMC5880626

[170] Sellmyer MA Richman SA Lohith K Hou C Weng C-C Mach RH. Imaging CAR T cell trafficking with eDHFR as a PET reporter gene. Mol Ther. (2020) 28:42–51. 10.1016/j.ymthe.2019.10.007 31668558 PMC6953896

[171] Massoud TF. Molecular imaging in living subjects: seeing fundamental biological processes in a new light. Genes Dev. (2003) 17:545–80. 10.1101/gad.1047403 12629038

[172] Simonetta F Alam IS Lohmeyer JK Sahaf B Good Z Chen W. Molecular imaging of chimeric antigen receptor T cells by ICOS-immunoPET. Clin Cancer Res. (2021) 27:1058–68. 10.1158/1078-0432.CCR-20-2770 33087332 PMC7887027

[173] Pichler BJ Kolb A Nägele T Schlemmer H-P. PET/MRI: paving the way for the next generation of clinical multimodality imaging applications. J Nucl Med. (2010) 51:333–6. 10.2967/jnumed.109.061853 20150252

[174] Yang C-T Ghosh KK Padmanabhan P Langer O Liu J Eng DNC. PET-MR and SPECT-MR multimodality probes: development and challenges. Theranostics. (2018) 8:6210–32. 10.7150/thno.26610 30613293 PMC6299694

[175] Patel P Baradaran H Delgado D Askin G Christos P John Tsiouris A. MR perfusion-weighted imaging in the evaluation of high-grade gliomas after treatment: a systematic review and meta-analysis. Neuro Oncol. (2017) 19:118–27. 10.1093/neuonc/now148 27502247 PMC5193025

[176] Hamieh M Dobrin A Cabriolu A van der Stegen SJC Giavridis T Mansilla-Soto J. CAR T cell trogocytosis and cooperative killing regulate tumour antigen escape. Nature. (2019) 568:112–6. 10.1038/s41586-019-1054-1 30918399 PMC6707377

[177] Hossain NM Dahiya S Le R Abramian AM Kong KA Muffly LS. Circulating tumor DNA assessment in patients with diffuse large B-cell lymphoma following CAR T-cell therapy. Leuk Lymphoma. (2019) 60:503–6. 10.1080/10428194.2018.1474463 29966461

[178] Bettegowda C Sausen M Leary RJ Kinde I Wang Y Agrawal N. Detection of circulating tumor DNA in early- and late-stage human malignancies. Sci Transl Med. (2014) 6:224ra24. 10.1126/scitranslmed.3007094 24553385 PMC4017867

[179] Faria G Silva E Da Fonseca C Quirico-Santos T. Circulating cell-free DNA as a prognostic and molecular marker for patients with brain tumors under perillyl alcohol-based therapy. Int J Mol Sci. (2018) 19:1610. 10.3390/ijms19061610 29848970 PMC6032335

[180] Piccioni DE Achrol AS Kiedrowski LA Banks KC Boucher N Barkhoudarian G. Analysis of cell-free circulating tumor DNA in 419 patients with glioblastoma and other primary brain tumors. CNS Oncol. (2019) 8:CNS34. 10.2217/cns-2018-0015 30855176 PMC6713031

[181] Mair R Mouliere F Smith CG Chandrananda D Gale D Marass F. Measurement of plasma cell-free mitochondrial tumor DNA improves detection of glioblastoma in patient-derived orthotopic xenograft models. Cancer Res. (2019) 79:220–30. 10.1158/0008-5472.CAN-18-0074 30389699 PMC6753020

[182] De Mattos-Arruda L Mayor R Ng CKY Weigelt B Martínez-Ricarte F Torrejon D. Cerebrospinal fluid-derived circulating tumour DNA better represents the genomic alterations of brain tumours than plasma. Nat Commun. (2015) 6:8839. 10.1038/ncomms9839 26554728 PMC5426516

[183] Bagley SJ O'Rourke DM. Clinical investigation of CAR T cells for solid tumors: lessons learned and future directions. Pharmacol Ther. (2020) 205:107419. 10.1016/j.pharmthera.2019.107419 31629009

[184] Namuduri M Brentjens RJ. Enhancing CAR T cell efficacy: the next step toward a clinical revolution? Expert Rev Hematol. (2020) 13:533–43. 10.1080/17474086.2020.1753501 32267181

[185] Morgan MA Schambach A. Chimeric antigen receptor T cells: extending translation from liquid to solid tumors. Hum Gene Ther. (2018) 29:1083–97. 10.1089/hum.2017.251 30156435

[186] Parker KR Migliorini D Perkey E Yost KE Bhaduri A Bagga P. Single-cell analyses identify brain mural cells expressing CD19 as potential off-tumor targets for CAR-T immunotherapies. Cell. (2020) 183:126–42.e17. 10.1016/j.cell.2020.08.022 32961131 PMC7640763

[187] Sottoriva A Spiteri I Piccirillo SGM Touloumis A Collins VP Marioni JC. Intratumor heterogeneity in human glioblastoma reflects cancer evolutionary dynamics. Proc Natl Acad Sci USA. (2013) 110:4009–14. 10.1073/pnas.1219747110 23412337 PMC3593922

[188] Patel AP Tirosh I Trombetta JJ Shalek AK Gillespie SM Wakimoto H. Single-cell RNA-seq highlights intratumoral heterogeneity in primary glioblastoma. Science. (2014) 344:1396–401. 10.1126/science.1254257 24925914 PMC4123637

[189] Inda M-M Bonavia R Mukasa A Narita Y Sah DWY Vandenberg S. Tumor heterogeneity is an active process maintained by a mutant EGFR-induced cytokine circuit in glioblastoma. Genes Dev. (2010) 24:1731–45. 10.1101/gad.1890510 20713517 PMC2922502

[190] Jubran MR Rubinstein AM Cojocari I Adejumobi IA Mogilevsky M Tibi S. Dissecting the role of crosstalk between glioblastoma subpopulations in tumor cell spreading. Oncogenesis. (2020) 9:11. 10.1038/s41389-020-0199-y 32024816 PMC7002777

[191] Johnson BE Mazor T Hong C Barnes M Aihara K McLean CY. Mutational analysis reveals the origin and therapy-driven evolution of recurrent glioma. Science. (2014) 343:189–93. 10.1126/science.1239947 24336570 PMC3998672

[192] Couturier CP Ayyadhury S Le PU Nadaf J Monlong J Riva G. Single-cell RNA-seq reveals that glioblastoma recapitulates a normal neurodevelopmental hierarchy. Nat Commun. (2020) 11:3406. 10.1038/s41467-020-17186-5 32641768 PMC7343844

[193] Hegde M Corder A Chow KKH Mukherjee M Ashoori A Kew Y. Combinational targeting offsets antigen escape and enhances effector functions of adoptively transferred T cells in glioblastoma. Mol Ther. (2013) 21:2087–101. 10.1038/mt.2013.185 23939024 PMC3831041

[194] Hegde M Mukherjee M Grada Z Pignata A Landi D Navai SA. Tandem CAR T cells targeting HER2 and IL13Rα2 mitigate tumor antigen escape. J Clin Invest. (2016) 126:3036–52. 10.1172/JCI83416 27427982 PMC4966331

[195] Krenciute G Prinzing BL Yi Z Wu M-F Liu H Dotti G. Transgenic expression of IL15 improves antiglioma activity of IL13Rα2-CAR T cells but results in antigen loss variants. Cancer Immunol Res. (2017) 5:571–81. 10.1158/2326-6066.CIR-16-0376 28550091 PMC5746871

[196] Wilkie S van Schalkwyk MCI Hobbs S Davies DM van der Stegen SJC Pereira ACP. Dual targeting of ErbB2 and MUC1 in breast cancer using chimeric antigen receptors engineered to provide complementary signaling. J Clin Immunol. (2012) 32:1059–70. 10.1007/s10875-012-9689-9 22526592

[197] Qin H Ramakrishna S Nguyen S Fountaine TJ Ponduri A Stetler-Stevenson M. Preclinical development of bivalent chimeric antigen receptors targeting both CD19 and CD22. Mol Ther Oncolytics. (2018) 11:127–37. 10.1016/j.omto.2018.10.006 30581986 PMC6300726

[198] Bielamowicz K Fousek K Byrd TT Samaha H Mukherjee M Aware N. Trivalent CAR T cells overcome interpatient antigenic variability in glioblastoma. Neuro Oncol. (2018) 20:506–18. 10.1093/neuonc/nox182 29016929 PMC5909636

[199] Goebeler M-E Bargou RC. T cell-engaging therapies—BiTEs and beyond. Nat Rev Clin Oncol. (2020) 17:418–34. 10.1038/s41571-020-0347-5 32242094

[200] Choi BD Yu X Castano AP Bouffard AA Schmidts A Larson RC. CAR-T cells secreting BiTEs circumvent antigen escape without detectable toxicity. Nat Biotechnol. (2019) 37:1049–58. 10.1038/s41587-019-0192-1 31332324

[201] Nduom EK Weller M Heimberger AB. Immunosuppressive mechanisms in glioblastoma. Neuro Oncol. (2015) 17:vii9–14. 10.1093/neuonc/nov151 26516226 PMC4625890

[202] Mangani D Weller M Roth P. The network of immunosuppressive pathways in glioblastoma. Biochem Pharmacol. (2017) 130:1–9. 10.1016/j.bcp.2016.12.011 28017775

[203] Grabowski MM Sankey EW Ryan KJ Chongsathidkiet P Lorrey SJ Wilkinson DS. Immune suppression in gliomas. J Neurooncol. (2021) 151:3–12. 10.1007/s11060-020-03483-y 32542437 PMC7843555

[204] DeCordova S Shastri A Tsolaki AG Yasmin H Klein L Singh SK. Molecular heterogeneity and immunosuppressive microenvironment in glioblastoma. Front Immunol. (2020) 11:1402. 10.3389/fimmu.2020.01402 32765498 PMC7379131

[205] Litak J Mazurek M Grochowski C Kamieniak P Roliński J. PD-L1/PD-1 axis in glioblastoma multiforme. Int J Mol Sci. (2019) 20:5347. 10.3390/ijms20215347 PMC6862444 31661771

[206] Zhu VF Yang J Lebrun DG Li M. Understanding the role of cytokines in glioblastoma multiforme pathogenesis. Cancer Lett. (2012) 316:139–50. 10.1016/j.canlet.2011.11.001 22075379

[207] Kesarwani P Prabhu A Kant S Chinnaiyan P. Metabolic remodeling contributes towards an immune-suppressive phenotype in glioblastoma. Cancer Immunol Immunother. (2019) 68:1107–20. 10.1007/s00262-019-02347-3 31119318 PMC6586493

[208] Markley JC Sadelain M. IL-7 and IL-21 are superior to IL-2 and IL-15 in promoting human T cell-mediated rejection of systemic lymphoma in immunodeficient mice. Blood. (2010) 115:3508–19. 10.1182/blood-2009-09-241398 20190192 PMC2867264

[209] He C Zhou Y Li Z Farooq MA Ajmal I Zhang H. Co-expression of IL-7 improves NKG2D-based CAR T cell therapy on prostate cancer by enhancing the expansion and inhibiting the apoptosis and exhaustion. Cancers (Basel). (2020) 12:1969. 10.3390/cancers12071969 32698361 PMC7409228

[210] Li Z Luo H Su J Sun R Sun Y Wang Y. Coexpression of IL-7 and CCL21 increases efficacy of CAR-T cells in solid tumors without requiring preconditioned lymphodepletion. Clin Cancer Res. (2020) 26:5494–5505. 10.1158/1078-0432.CCR-20-0777 32816947

[211] Zhang L Morgan RA Beane JD Zheng Z Dudley ME Kassim SH. Tumor-infiltrating lymphocytes genetically engineered with an inducible gene encoding interleukin-12 for the immunotherapy of metastatic melanoma. Clin Cancer Res. (2015) 21:2278–88. 10.1158/1078-0432.CCR-14-2085 25695689 PMC4433819

[212] Kueberuwa G Kalaitsidou M Cheadle E Hawkins RE Gilham DE. CD19 CAR T cells expressing IL-12 eradicate lymphoma in fully lymphoreplete mice through induction of host immunity. Mol Ther Oncolytics. (2018) 8:41–51. 10.1016/j.omto.2017.12.003 29367945 PMC5772011

[213] Liu Y Di S Shi B Zhang H Wang Y Wu X. Armored inducible expression of IL-12 enhances antitumor activity of glypican-3-targeted chimeric antigen receptor-engineered T cells in hepatocellular carcinoma. J Immunol. (2019) 203:198–207. 10.4049/jimmunol.1800033 31142602

[214] Hu B Ren J Luo Y Keith B Young RM Scholler J. Augmentation of antitumor immunity by human and mouse CAR T cells secreting IL-18. Cell Rep. (2017) 20:3025–33. 10.1016/j.celrep.2017.09.002 28954221 PMC6002762

[215] Chmielewski M Abken H. CAR T cells releasing IL-18 convert to T-bethigh foxo1low effectors that exhibit augmented activity against advanced solid tumors. Cell Rep. (2017) 21:3205–19. 10.1016/j.celrep.2017.11.063 29241547

[216] Zimmermann K Kuehle J Dragon AC Galla M Kloth C Rudek LS. Design and characterization of an “All-in-One” lentiviral vector system combining constitutive anti-GD2 CAR expression and inducible cytokines. Cancers (Basel). (2020) 12:375. 10.3390/cancers12020375 32041222 PMC7072617

[217] Spolski R Leonard WJ. Interleukin-21: a double-edged sword with therapeutic potential. Nat Rev Drug Discov. (2014) 13:379–95. 10.1038/nrd4296 24751819

[218] Singh H Figliola MJ Dawson MJ Huls H Olivares S Switzer K. Reprogramming CD19-specific T cells with IL-21 signaling can improve adoptive immunotherapy of B-lineage malignancies. Cancer Res. (2011) 71:3516–27. 10.1158/0008-5472.CAN-10-3843 21558388 PMC3096697

[219] Ma X Shou P Smith C Chen Y Du H Sun C. Interleukin-23 engineering improves CAR T cell function in solid tumors. Nat Biotechnol. (2020) 38:448–59. 10.1038/s41587-019-0398-2 32015548 PMC7466194

[220] Chmielewski M Abken H. TRUCKs: the fourth generation of CARs. Expert Opin Biol Ther. (2015) 15:1145–54. 10.1517/14712598.2015.1046430 25985798

[221] Labanieh L Majzner RG Mackall CL. Programming CAR-T cells to kill cancer. Nat Biomed Eng. (2018) 2:377–91. 10.1038/s41551-018-0235-9 31011197

[222] Rice J Nagle S Randall J Hinson HE. Chimeric antigen receptor T cell-related neurotoxicity: mechanisms, clinical presentation, and approach to treatment. Curr Treat Options Neurol. (2019) 21:40. 10.1007/s11940-019-0580-3 31327064

[223] Gust J Ponce R Liles WC Garden GA Turtle CJ. Cytokines in CAR T cell-associated neurotoxicity. Front Immunol. (2020) 11:577027. 10.3389/fimmu.2020.577027 33391257 PMC7772425

[224] Klemm F Maas RR Bowman RL Kornete M Soukup K Nassiri S. Interrogation of the microenvironmental landscape in brain tumors reveals disease-specific alterations of immune cells. Cell. (2020) 181:1643–60.e17. 10.1016/j.cell.2020.05.007 32470396 PMC8558904

[225] Akkari L Bowman RL Tessier J Klemm F Handgraaf SM de Groot M. Dynamic changes in glioma macrophage populations after radiotherapy reveal CSF-1R inhibition as a strategy to overcome resistance. Sci Transl Med. (2020) 12:eaaw7843. 10.1126/scitranslmed.aaw7843 32669424

[226] Liu X Ranganathan R Jiang S Fang C Sun J Kim S. A chimeric switch-receptor targeting PD1 augments the efficacy of second-generation CAR T cells in advanced solid tumors. Cancer Res. (2016) 76:1578–90. 10.1158/0008-5472.CAN-15-2524 26979791 PMC4800826

[227] Mohammed S Sukumaran S Bajgain P Watanabe N Heslop HE Rooney CM. Improving chimeric antigen receptor-modified T cell function by reversing the immunosuppressive tumor microenvironment of pancreatic cancer. Mol Ther. (2017) 25:249–58. 10.1016/j.ymthe.2016.10.016 28129119 PMC5363304

[228] Wang Y Jiang H Luo H Sun Y Shi B Sun R. An IL-4/21 inverted cytokine receptor improving CAR-T cell potency in immunosuppressive solid-tumor microenvironment. Front Immunol. (2019) 10:1691. 10.3389/fimmu.2019.01691 31379876 PMC6658891

[229] Chen N Morello A Tano Z Adusumilli PS. CAR T-cell intrinsic PD-1 checkpoint blockade: a two-in-one approach for solid tumor immunotherapy. Oncoimmunology. (2017) 6:e1273302. 10.1080/2162402X.2016.1273302 28344886 PMC5353939

[230] Kloss CC Lee J Zhang A Chen F Melenhorst JJ Lacey SF. Dominant-negative TGF-β receptor enhances PSMA-targeted human CAR T cell proliferation and augments prostate cancer eradication. Mol Ther. (2018) 26:1855–66. 10.1016/j.ymthe.2018.05.003 29807781 PMC6037129

[231] Tan AHJ Vinanica N Campana D. Chimeric antigen receptor-T cells with cytokine neutralizing capacity. Blood Adv. (2020) 4:1419–31. 10.1182/bloodadvances.2019001287 32271901 PMC7160280

[232] Gao Q Dong X Xu Q Zhu L Wang F Hou Y. Therapeutic potential of CRISPR/Cas9 gene editing in engineered T-cell therapy. Cancer Med. (2019) 8:4254–64. 10.1002/cam4.2257 31199589 PMC6675705

[233] Hu B Zou Y Zhang L Tang J Niedermann G Firat E. Nucleofection with plasmid DNA for CRISPR/Cas9-mediated inactivation of programmed cell death protein 1 in CD133-specific CAR T cells. Hum Gene Ther. (2019) 30:446–58. 10.1089/hum.2017.234 29706119

[234] Choi BD Yu X Castano AP Darr H Henderson DB Bouffard AA. CRISPR-Cas9 disruption of PD-1 enhances activity of universal EGFRvIII CAR T cells in a preclinical model of human glioblastoma. J Immunother cancer. (2019) 7:304. 10.1186/s40425-019-0806-7 31727131 PMC6857271

[235] Chédeville AL Lourdusamy A Monteiro AR Hill R Madureira PA. Investigating glioblastoma response to hypoxia. Biomedicines. (2020) 8:310. 10.3390/biomedicines8090310 PMC7555589 32867190

[236] Juillerat A Marechal A Filhol JM Valogne Y Valton J Duclert A. An oxygen sensitive self-decision making engineered CAR T-cell. Sci Rep. (2017) 7:39833. 10.1038/srep39833 28106050 PMC5247770

[237] Vuillefroy de Silly R Dietrich P-Y Walker PR. Hypoxia and antitumor CD8+ T cells: an incompatible alliance? Oncoimmunology. (2016) 5:e1232236. 10.1080/2162402X.2016.1232236 28123871 PMC5214994

[238] Robertson FL Marqués-Torrejón M-A Morrison GM Pollard SM. Experimental models and tools to tackle glioblastoma. Dis Model Mech. (2019) 12:dmm040386. 10.1242/dmm.040386 31519690 PMC6765190

[239] Lee J Kotliarova S Kotliarov Y Li A Su Q Donin NM. Tumor stem cells derived from glioblastomas cultured in bFGF and EGF more closely mirror the phenotype and genotype of primary tumors than do serum-cultured cell lines. Cancer Cell. (2006) 9:391–403. 10.1016/j.ccr.2006.03.030 16697959

[240] Azzarelli R. Organoid models of glioblastoma to study brain tumor stem cells. Front cell Dev Biol. (2020) 8:220. 10.3389/fcell.2020.00220 32373607 PMC7176979

[241] Patrizii M Bartucci M Pine SR Sabaawy HE. Utility of glioblastoma patient-derived orthotopic xenografts in drug discovery and personalized therapy. Front Oncol. (2018) 8:23. 10.3389/fonc.2018.00023 29484285 PMC5816058

[242] Valtorta S Salvatore D Rainone P Belloli S Bertoli G Moresco RM. Molecular and cellular complexity of glioma. Focus on tumour microenvironment and the use of molecular and imaging biomarkers to overcome treatment resistance. Int J Mol Sci. (2020) 21:5631. 10.3390/ijms21165631 32781585 PMC7460665

[243] Jiang Z Jiang X Chen S Lai Y Wei X Li B. Anti-GPC3-CAR T cells suppress the growth of tumor cells in patient-derived xenografts of hepatocellular carcinoma. Front Immunol. (2017) 7:690. 10.3389/fimmu.2016.00690 28123387 PMC5225101

[244] Teng R Zhao J Zhao Y Gao J Li H Zhou S. Chimeric antigen receptor–modified T cells repressed solid tumors and their relapse in an established patient-derived colon carcinoma xenograft model. J Immunother. (2019) 42:33–42. 10.1097/CJI.0000000000000251 30586347 PMC6382056

[245] Smith KS Xu K Mercer KS Boop F Klimo P DeCupyere M. Patient-derived orthotopic xenografts of pediatric brain tumors: a St. Jude resource. Acta Neuropathol. (2020) 140:209–25. 10.1007/s00401-020-02171-5 32519082 PMC7360541

[246] Zhang H Qi L Du Y Huang LF Braun FK Kogiso M. Patient-derived orthotopic xenograft (PDOX) mouse models of primary and recurrent meningioma. Cancers (Basel). (2020) 12:1478. 10.3390/cancers12061478 32517016 PMC7352400

[247] Ben-David U Ha G Tseng Y-Y Greenwald NF Oh C Shih J. Patient-derived xenografts undergo mouse-specific tumor evolution. Nat Genet. (2017) 49:1567–75. 10.1038/ng.3967 28991255 PMC5659952

[248] Villacorta-Martin C Craig AJ Villanueva A. Divergent evolutionary trajectories in transplanted tumor models. Nat Genet. (2017) 49:1565–6. 10.1038/ng.3983 29074950

[249] Marques-Torrejon MA Gangoso E Pollard SM. Modelling glioblastoma tumour-host cell interactions using adult brain organotypic slice co-culture. Dis Model Mech. (2018) 11:dmm031435. 10.1242/dmm.031435 29196443 PMC5894940

[250] Shultz LD Schweitzer PA Christianson SW Gott B Schweitzer IB Tennent B. Multiple defects in innate and adaptive immunologic function in NOD/LtSz-scid mice. J Immunol. (1995) 154:180–91. 7995938

[251] Halkias J Yen B Taylor KT Reinhartz O Winoto A Robey EA. Conserved and divergent aspects of human T-cell development and migration in humanized mice. Immunol Cell Biol. (2015) 93:716–26. 10.1038/icb.2015.38 25744551 PMC4575952

[252] Wen H Qu Z Yan Y Pu C Wang C Jiang H. Preclinical safety evaluation of chimeric antigen receptor-modified T cells against CD19 in NSG mice. Ann Transl Med. (2019) 7:735. 10.21037/atm.2019.12.03 32042751 PMC6990014

[253] Shultz LD Brehm MA Garcia-Martinez JV Greiner DL. Humanized mice for immune system investigation: progress, promise and challenges. Nat Rev Immunol. (2012) 12:786–98. 10.1038/nri3311 23059428 PMC3749872

[254] Mhaidly R Verhoeyen E. Humanized mice are precious tools for preclinical evaluation of CAR T and CAR NK cell therapies. Cancers (Basel). (2020) 12:1915. 10.3390/cancers12071915 32679920 PMC7409195

[255] Coughlan AM Harmon C Whelan S O'Brien EC O'Reilly VP Crotty P. Myeloid engraftment in humanized mice: impact of granulocyte-colony stimulating factor treatment and transgenic mouse strain. Stem Cells Dev. (2016) 25:530–41. 10.1089/scd.2015.0289 26879149

[256] Walsh NC Kenney LL Jangalwe S Aryee K-E Greiner DL Brehm MA. Humanized mouse models of clinical disease. Annu Rev Pathol Mech Dis. (2017) 12:187–215. 10.1146/annurev-pathol-052016-100332 PMC5280554 27959627

[257] Wunderlich M Chou F-S Sexton C Presicce P Chougnet CA Aliberti J. Improved multilineage human hematopoietic reconstitution and function in NSGS mice. PLoS ONE. (2018) 13:e0209034. 10.1371/journal.pone.0209034 30540841 PMC6291127

[258] Verma B Wesa A. Establishment of humanized mice from peripheral blood mononuclear cells or cord blood CD34+ hematopoietic stem cells for immune-oncology studies evaluating new therapeutic agents. Curr Protoc Pharmacol. (2020) 89:e77. 10.1002/cpph.77 32453514

[259] Koehler JW Miller AD Miller CR Porter B Aldape K Beck J. A revised diagnostic classification of canine glioma: towards validation of the canine glioma patient as a naturally occurring preclinical model for human glioma. J Neuropathol Exp Neurol. (2018) 77:1039–54. 10.1093/jnen/nly085 30239918 PMC6181180

[260] Migliorini D Mason NJ Posey AD. Keeping the engine running: the relevance and predictive value of preclinical models for CAR-T cell development. ILAR J. (2018) 59:276–85. 10.1093/ilar/ilz009 31095687

[261] Thommen DS Schumacher TN. T cell dysfunction in cancer. Cancer Cell. (2018) 33:547–62. 10.1016/j.ccell.2018.03.012 29634943 PMC7116508

[262] Fu W Wang W Li H Jiao Y Huo R Yan Z. Single-cell atlas reveals complexity of the immunosuppressive microenvironment of initial and recurrent glioblastoma. Front Immunol. (2020) 11:835. 10.3389/fimmu.2020.00835 32457755 PMC7221162

