# Supplementary material for: Allogeneic CAR T Cells: An Alternative to Overcome Challenges of CAR T Cell Therapy in Glioblastoma
Source: Front Immunol. 2021 Mar 3;12:640082. doi: 10.3389/fimmu.2021.640082 (PMC7966522; doi:10.3389/fimmu.2021.640082)
Supplement: Supplementary file 1 [file Table_1.DOCX]

Supplementary Material

# Supplementary Table 1. Clinical trials using CAR T cells against GBM

| CT Identifier (related papers) | Phase | Disease | Target | Sponsor | Start time  /Completion | Status  / Number enrolled | Primary outcome | Clinical Results |
| --- | --- | --- | --- | --- | --- | --- | --- | --- |
| NCT00730613 (1) | I | Brain and Central Nervous System Tumors | IL13Rα2 | City of Hope Medical Center (USA) | 2002/2011 | Completed /3 | Feasibility Safety | 2 showed no recurrence |
| NCT01109095 (2,3) | I | GBM | HER2 | Baylor College of Medicine (USA) | 2010/2018 | Completed /16 | Number of subjects with DLT after CTL infusion | 1 PR, 7 SD, 8 PD |
| NCT01454596 (4) | I/II | Malignant glioma, GBM, brain cancer, Gliosarcoma | EGFRvIII | National Cancer Institute (USA) | 2012/2019 | Completed /18 | TRAE, PFS | none |
| NCT02209376 (5,6) | I | Residual or Recurrent EGFRvIII+ glioma | EGFRvIII | University of Pennsylvania (USA) | 2014/2018 | Terminated /10 | TRAE | 9 SD, 1 PD |
| NCT02208362 (7) | I | Malignant Glioma, Refractory or Recurrent Brain Neoplasm, GBM | IL13Rα2 | City of Hope Medical Center (USA) | 2015/2021* | Recruiting /92* | TRAE, DLT | 1 CR for 7.5 months |
| NCT02844062 | I | GBM | EGFRvIII | Beijing Sanbo Brain Hospital (China) | 2016/2019* | Unknown /20* | TRAE | n/a |
| NCT02937844 | I | GBM | PD-L1 | Beijing Sanbo Brain Hospital (China) | 2016/2019* | Unknown /20* | TRAE | n/a |
| NCT02442297 | I | Brain Tumor, Recurrent and Refractory | HER2 | Baylor College of Medicine (USA) | 2016/2031* | Recruiting /28* | DLT | n/a |
| NCT02664363 (8) | I | GBM and gliosarcoma | EGFRvIII | Duke University (USA) | 2017/2019 | Terminated /3 | MTD | n/a |
| NCT03383978 | I | GBM | HER2 | Johann Wolfgang Goethe University Hospital (Germany) | 2017/2020* | Recruiting /30* | TRAE, MTD, MFD, persistence and cytokine profile | n/a |
| NCT03170141 | I | GBM | GBM antigens not specified | Shenzhen Geno-Immune Medical Institute (China) | 2017/2020* | Enrolling by invitation /20* | TRAE | n/a |
| NCT03392545 | I | High Grade Glioma, GBM, Glioma of Brainstem | n/a | Beijing Tiantan Hospital (China) | 2018/2020* | Recruiting /30* | TRAE | n/a |
| NCT03389230 | I | GBM, Malignant Glioma, Recurrent or Refractory Glioma, WHO Grade III Glioma | HER2 | City of Hope Medical Center (USA) | 2018/2021* | Recruiting /42* | TRAE, DLT | n/a |
| NCT03283631 | I | Recurrent GBM and gliosarcoma | EGFRvIII | Duke University (USA) | 2018/2022* | Suspended /24* | MTD | n/a |
| NCT04003649 | I | Recurrent or refractory GBM | IL13Rα2 | City of Hope Medical Center (USA) | 2019/2022* | Recruiting /60* | TRAE, DLT, feasibility of complete 4 cycles, OS | n/a |
| NCT04045847 | I | Recurrent GBM CD147+ | CD147 | Xijing Hospital (China) | 2019/2022* | Recruiting /31* | TRAE | n/a |
| NCT03726515 | I | GBM | EGFRvIII | University of Pennsylvania (USA) | 2019/2034* | Active, not recruiting /7 | TRAE | n/a |
| NCT04385173 | I | Recurrent or refractory GBM | B7-H3 | Second Affiliated Hospital, Zhejiang University (China) | 2020*/2022* | Recruiting /12* | TRAE, MTD, OS, PFS | n/a |
| NCT04214392 | I | Recurrent GBM, malignant glioma and WHO grade II and II glioma | MMP-2 | City of Hope Medical Center (USA) | 2020/2023* | Recruiting /36* | DLT | n/a |
| NCT04270461 | I | Hepatocellular Carcinoma, GBM, Medulloblastoma Colon Cancer | NKG2DL | Jiujiang University Affiliated Hospital (China) | 2020*/2023* | Not yet recruiting /10* | TRAE (severe CRS) and copy numbers of CAR | n/a |
| NCT04077866 | I/II | Recurrent or refractory GBM | B7-H3 | Second Affiliated Hospital, Zhejiang University (China) | 2022*/2024* | Recruiting /40* | OS | n/a |

Legend:

*: estimated; n/a: not available; CRS: cytokine release syndrome; DLT: dose-limiting toxicity; MTD: maximum tolerated dose; OS: overall survival; PFS: progression-free survival; TRAE: treatment-related adverse events; CR: complete response; PR: partial response; SD: stable disease.

**Publications related to the clinical trials:**

1. Brown CE, Badie B, Barish ME, Weng L, Ostberg JR, Chang W-C, Naranjo A, Starr R, Wagner J, Wright C, et al. Bioactivity and Safety of IL13Rα2-Redirected Chimeric Antigen Receptor CD8+ T Cells in Patients with Recurrent Glioblastoma. *Clin Cancer Res* (2015) **21**:4062–72. doi:10.1158/1078-0432.CCR-15-0428

2. Badhiwala J, Decker WK, Berens ME, Bhardwaj RD. Clinical trials in cellular immunotherapy for brain/CNS tumors. *Expert Rev Neurother* (2013) **13**:405–424. doi:10.1586/ern.13.23

3. Ahmed N, Brawley V, Hegde M, Bielamowicz K, Kalra M, Landi D, Robertson C, Gray TL, Diouf O, Wakefield A, et al. HER2-Specific Chimeric Antigen Receptor-Modified Virus-Specific T Cells for Progressive Glioblastoma: A Phase 1 Dose-Escalation Trial. *JAMA Oncol* (2017) **3**:1094–1101. doi:10.1001/jamaoncol.2017.0184

4. Morgan RA, Johnson LA, Davis JL, Zheng Z, Woolard KD, Reap EA, Feldman SA, Chinnasamy N, Kuan C-T, Song H, et al. Recognition of Glioma Stem Cells by Genetically Modified T Cells Targeting EGFRvIII and Development of Adoptive Cell Therapy for Glioma. *Hum Gene Ther* (2012) **23**:1043–1053. doi:10.1089/hum.2012.041

5. Johnson LA, Scholler J, Ohkuri T, Kosaka A, Patel PR, McGettigan SE, Nace AK, Dentchev T, Thekkat P, Loew A, et al. Rational development and characterization of humanized anti-EGFR variant III chimeric antigen receptor T cells for glioblastoma. *Sci Transl Med* (2015) **7**:275ra22. doi:10.1126/scitranslmed.aaa4963

6. O’Rourke DM, Nasrallah MP, Desai A, Melenhorst JJ, Mansfield K, Morrissette JJD, Martinez-Lage M, Brem S, Maloney E, Shen A, et al. A single dose of peripherally infused EGFRvIII-directed CAR T cells mediates antigen loss and induces adaptive resistance in patients with recurrent glioblastoma. *Sci Transl Med* (2017) **9**:eaaa0984. doi:10.1126/scitranslmed.aaa0984

7. Brown CE, Alizadeh D, Starr R, Weng L, Wagner JR, Naranjo A, Ostberg JR, Blanchard MS, Kilpatrick J, Simpson J, et al. Regression of Glioblastoma after Chimeric Antigen Receptor T-Cell Therapy. *N Engl J Med* (2016) **375**:2561–9. doi:10.1056/NEJMoa1610497

8. Suryadevara CM, Desai R, Abel ML, Riccione KA, Batich KA, Shen SH, Chongsathidkiet P, Gedeon PC, Elsamadicy AA, Snyder DJ, et al. Temozolomide lymphodepletion enhances CAR abundance and correlates with antitumor efficacy against established glioblastoma. *Oncoimmunology* (2018) **7**:e1434464. doi:10.1080/2162402X.2018.1434464
